# Patterns and Direct/Indirect Signaling Pathways in Cardiovascular System in the Condition of Transient Increase of NO

**DOI:** 10.1155/2020/6578213

**Published:** 2020-05-27

**Authors:** Anton Misak, Lucia Kurakova, Andrea Berenyiova, Lenka Tomasova, Marian Grman, Sona Cacanyiova, Karol Ondrias

**Affiliations:** ^1^Institute of Clinical and Translational Research, Biomedical Research Center, Slovak Academy of Sciences, Dubravska cesta 9, 845 05 Bratislava, Slovakia; ^2^Department of Pharmacology and Toxicology, Faculty of Pharmacy, Comenius University, Odbojarov 10, 832 32 Bratislava, Slovakia; ^3^Institute of Normal and Pathological Physiology, Centre of Experimental Medicine, Slovak Academy of Sciences, Dubravska cesta 9, 841 04 Bratislava, Slovakia

## Abstract

**Aim:**

To study “patterns” and connections of signaling pathways derived from the rat arterial pulse waveform (APW) under the condition of transient NO increase.

**Methods and Results:**

The right jugular vein of anesthetized Wistar rats was cannulated for administration of NO donor S-nitrosoglutathione. The left carotid artery was cannulated to detect APW. From rat APW, 35 hemodynamic parameters (HPs) and several their crossrelationships were evaluated. We introduced a new methodology to study “patterns” and connections of different signaling pathways, which are suggested from hysteresis and nonhysteresis crossrelationships of 35 rat HPs. Here, we show parallel time-dependent patterns of 35 HPs and some of their crossrelationships under the condition of transient increase of NO bioavailability by administration of S-nitrosoglutathione. Approximate nonhysteresis relationships were observed between systolic blood pressure and at least 11 HPs suggesting that these HPs, i.e., their signaling pathways, responding to NO concentration, are directly connected. Hysteresis relationships were observed between systolic blood pressure and at least 14 HPs suggesting that the signaling pathways of these HPs are indirectly connected. Totally, from the crossrelationships of 35 HPs, one can obtain 595 “patterns” and indication of direct or indirect connections between the signaling pathways.

**Conclusion:**

We described the procedure leading virtually to 595 relationships, from which “patterns” for transient NO increase and direct or indirect connections of signaling pathways can be suggested. From a clinical perspective, this approach may be used in animal models and in humans to create a data bank of patterns of crossrelationships of HPs for different cardiovascular conditions. By comparison with unknown patterns of studied APW with the data bank patterns, it would be possible to determine cardiovascular conditions of the studied subject from the recorded arterial blood pressure. Additionally, it can help to find molecular mechanism of particular (patho-) physiological conditions or drug action and may have predictive or diagnostic value.

## 1. Introduction

Nitric oxide (NO) is a gaseous free radical occurring also in biological systems, where it is produced by the nitric oxide synthase (NOS) family. The NO/NOS system exerts a broad spectrum of signaling functions including modulation of cardiovascular pathophysiological conditions [1–6]. The NO donor, S-nitrosoglutathione (GSNO), has been thought to be a store of NO and may have potentially therapeutic beneficial effects in cases of NO deficiency [7]. Several signaling pathways were explored to understand biological effects of NO [5, 8, 9].

The information obtained from the shape of an arterial pulse waveform (APW) analysis can provide insight into many diseases. Several APW parameters were shown to be useful for characterization of the cardiovascular system [10–19]. Recently, we introduced measurement of 35 parallel time-dependent rat hemodynamic parameters (HPs) from which crossrelationships can be evaluated [20]. The present work is also based on the hypothesis that it is possible to characterize the cardiovascular system in many pathophysiological conditions just from the detailed shape of APW. The HPs and their crossrelationships may provide “patterns” for particular cardiovascular conditions, as we obtained for conditions of prolonged decreased NO bioavailability [20].

The molecular mechanism of the pathophysiology of living organisms, effect of drugs, signaling pathways, and their connections are extensively studied [6, 21–23]. To address this problem in cardiovascular signaling, we looked for specific “patterns” of time dependence of 35 HPs and some of their crossrelationships in the condition of transient increase of NO bioavailability. Since effects of NO were transient, it was possible to study nonhysteresis/hysteresis time-dependence changes of 35 HPs. From the crossrelationships, the “patterns” of direct or indirect signaling pathways were suggested.

## 2. Methods

### 2.1. Ethical Approval

All procedures were approved by the State Veterinary and Food Administration of the Slovak Republic (C.k. Ro 3123/17-221) according to the guidelines from Directive 2010/63/EU of the European Parliament. The procuration of animals, the husbandry, and the experiments conformed to the European Convention for the Protection of Vertebrate Animals used for Experimental and other Scientific Purposes (Council of Europe No 123, Strasbourg 1985). Experiments were carried out according to the guidelines laid down by the animal welfare committee of the Biomedical Research Center, Slovak Academy of Sciences, Bratislava, and conformed to the principles and regulations, as described in the editorial by Grundy [24].

### 2.2. Animals, APW Measurement, and Data Evaluation

**Figure 1:**
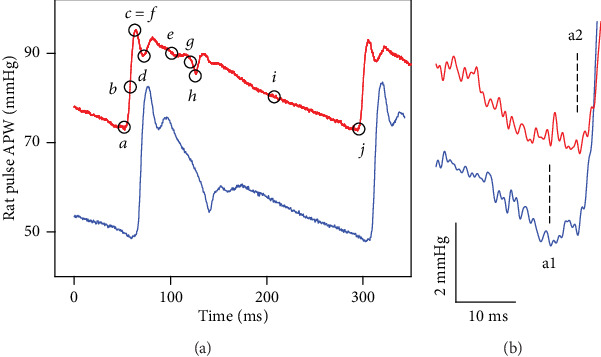
The left common carotid artery pulse waveform (APW) in the anesthetized rat. (a) Control APW (red) with marked ten points (black) before GSNO administration. APW recorded 15 s after GSNO (32 nmol kg^−1^) i.v. administration (blue). (b) Fluctuation of minimum diastolic BP, point *a* (a1 or a2).

Male Wistar rats (*n* = 10; 340 ± 40 g) were purchased from the Department of Toxicology and Laboratory Animal Breeding at Dobra Voda, Slovak Academy of Sciences, Slovakia. The rats were housed under a 12 h light-12 h dark cycle, at a constant humidity (45-65%) and temperature (20-22°C), with free access to standard laboratory rat chow and drinking water. The veterinary nursing care was provided by the Central Animal Housing Facility of Pavilion of Medical Sciences (registration number SK UCH 01017). Rats were anesthetized with Zoletil 100 (tiletamine+zolazepam, 80 mg kg^−1^, i.p.) and xylazine (5 mg kg^−1^, i.p.). The animals were under anesthesia during the whole experiment and were euthanized with an overdose of Zoletil/xylazine via the jugular vein at the end of the surgical procedure. APW continuous measurement, data analysis, and processing were done essentially as described in [20]. Basically, the right jugular vein of the anesthetized rat was cannulated for administration of experimental substances or medication. Chirurgical operation started on average ten minutes after administration of anesthesia and lasted for 18 ± 4 min. Next, 10-15 min was needed to stabilize systolic BP. After stabilization of the systolic blood pressure (BP), the NO donor GSNO (32 nmol kg^−1^), prepared in 0.9% saline solution, was administered into the right jugular vein (500 *μ*l kg^−1^) over 15 s period; thus, the first GSNO administration was 40-50 min after anesthesia administration. The left common carotid artery (*arteria carotis communis*) was cannulated to insert the fiber-optic microcatheter pressure transducers (FISO LS 2F Harvard Apparatus, USA) connected to the FISO Series Signal Conditioners, and the EVO Chassis was used to measure APW. The analogue signal was filtered by low-pass filter 2.5 kHz, digitalized at 10 kHz, and analyzed by an application written in MATLAB (the MathWorks, Inc., Natick, MA, USA) to identify and analyze ten points (*a*–*j*) of APW, which are marked in Figure 1(a). The definition and abbreviation of the 35 HP parameters calculated from APW points are stated in the following section. Since points *a* and *j* fluctuated with time intervals ~5-10 ms and ~1 mmHg BP, respectively (Figure 1(b)), when it was necessary, the data were filtered to average the fluctuation.

### 2.3. Description of 35 HPs

**Figure 2:**
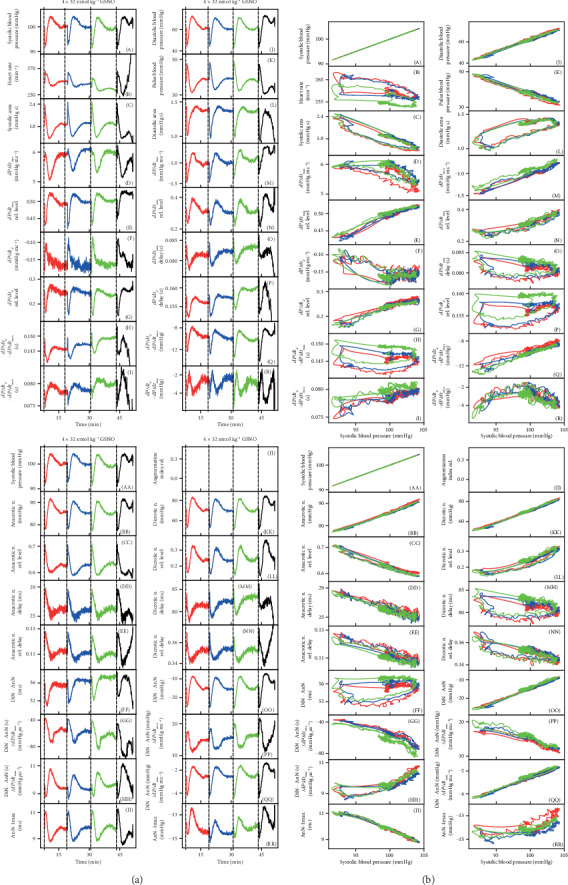
(a) Time-dependent changes of HPs of anesthetized rat after four subsequent i.v. bolus administrations of 32 nmol kg^−1^ GSNO (marked by dash lines). Each administration and recording HP has a different color. The red line starts 3 s before GSNO administration. (b) Relationships of HPs to systolic BP after the first three administrations of 32 nmol kg^−1^ GSNO. The data and colors were taken from (a).

Ten points *a*–*j* (in italicized letters) are from Figure 1(a), and they mark the values of BP and time that are used to define (calculate) specific HP. The letters in parentheses (A) to (R) and (AA) to (RR) refer to graphs in the figures, and each one represents HP; for example, (B) in Figure 2(a) represents heart rate. For more details, see [20].

- Systolic blood pressure in mmHg; point *c* or *f*.
- Heart rate in min^−1^; 60/(*j* − *a*); (*j* − *a*) represents the time interval between *a* and *j*; *a* and *j* are two reference points to diastolic BP value
- Systolic area in mmHg s; integral BP of *a* to *h*; *h* refers to BP at the dicrotic notch (dicrotic BP)
- *dP*/*dt*_max_ in mmHg ms^−1^; maximum derivative at point *b*; *P* is BP in mmHg
- *dP*/*dt*_max_ relative level; relative level (RL for short) of point *b*; (*b* − *a*)/(*c* (or *f*) − *a*) in mmHg/mmHg (dimensionless)
- *dP*/*dt*_*d*_ in mmHg ms^−1^; negative maximum derivative at point *i*; point *i* is the BP at the middle of the time interval between *h* and *j*
- *dP*/*dt*_*d*_ relative level, relative level of point *i*; (*i* − *a*)/(*c* (or *f*) − *a*) in mmHg/mmHg (dimensionless)
- *dP*/*dt*_*d*_ − *dP*/*dt*_max_ in s; time interval between *b* and *i*, *dP*/*dt*_*d*_ − *dP*/*dt*_max_ = (*i* − *b*)
- *dP*/*dt*_*d*_ − *dP*/*dt*_min_ in s; time interval between *g* and *i*, *dP*/*dt*_*d*_ − *dP*/*dt*_min_ = (*i* − *g*); *dP*/*dt*_min_ is the negative maximum derivative at the point *g*
- Diastolic blood pressure in mmHg; the point *a* or *j*
- Pulse BP in mmHg; (*c* − *a*) or (*f* − *a*)
- Diastolic area in mmHg s; integral BP of *h* to *j*
- *dP*/*dt*_min_ in mmHg ms^–1^; *dP*/*dt*_min_ is the maximum negative derivative at the point *g*
- *dP*/*dt*_min_ relative level, relative level of point *g*; (*g* − *a*)/(*c* (or *f*) − *a*) in mmHg/mmHg (dimensionless)
- *dP*/*dt*_min_ delay in s; delay in s of point *g*; (*g* − *a*) time interval between *a* and *g*
- *dP*/*dt*_*d*_ delay in s; delay in s of point *i*; (*i* − *a*) time interval between *a* and *i*
- *dP*/*dt*_*d*_ − *dP*/*dt*_max_ in mmHg; (*i* − *b*) BP difference between *b* and *i*
- *dP*/*dt*_*d*_ − *dP*/*dt*_min_ in mmHg; (*i* − *g*) BP difference between *g* and *i*
- Systolic blood pressure in mmHg; point *c* or *f*. Plot (AA) is the same as (A)
- Anacrotic notch in mmHg; BP at point *d*
- Anacrotic notch relative level; relative level of point *d*; (*d* − *a*)/(*c* (or *f*) − *a*) in mmHg/mmHg (dimensionless)
- Anacrotic notch delay in ms; delay in ms of point *d*; (*d* − *a*) time interval between *a* and *d*
- Anacrotic notch relative delay; relative delay (RD for short) of point *d*; (*d* − *a*)/(*j* − *a*) in ms/ms (dimensionless)
- [Dicrotic notch (DiN) in s] − [anacrotic notch (AnN) in s] in s; (*h* − *d*) time interval between *d* and *h*
- [(DiN − AnN) in s]/[*dP*/*dt*_min_ in mmHg *μ*s^−1^] in s/mmHg *μ*s^−1^; (*h* − *d*)/*g*
- [(DiN − AnN) in s]/[*dP*/*dt*_max_ in mmHg *μ*s^−1^] in s/mmHg *μ*s^−1^; (*h* − *d*)/*b*
- [AnN in ms] − [1max (point *c* or the 1st maximum) in ms] in ms; (*d* − *c*) time interval between *c* and *d*
- Augmentation index relative; (*f* − *c*)/(*f* − *a*) in mmHg/mmHg (dimensionless)
- Dicrotic notch in mmHg; BP at the point *h*
- Dicrotic notch relative level; relative level of point *h*; (*h* − *a*)/(*c* (or *f*) − *a*) in mmHg/mmHg (dimensionless)
- Dicrotic notch delay in ms, delay in ms of point *h*; (*h* − *a*) time interval between *a* and *h*
- Dicrotic notch relative delay; relative delay of point *h*; (*h* − *a*)/(*j* − *a*); in ms/ms (dimensionless)
- [DiN in mmHg] − [AnN in mmHg] in mmHg; (h − d) BP difference between *d* and *h*
- [(DiN − AnN) in mmHg]/[*dP*/*dt*_min_ in mmHg ms^−1^] in mmHg/mmHg ms^−1^; (*h* − *d*)/*g*
- [(DiN − AnN) in mmHg]/[*dP*/*dt*_max_ in mmHg ms^−1^] in mmHg/mmHg ms^−1^; (*h* − *d*)/*b*
- [AnN in mmHg] − [1max (point *c* or the 1st maximum) in mmHg] in mmHg; (*d* − *c*) BP difference between *c* and *d*

## 3. Results

### 3.1. Crossrelationships between HPs after Repeated GSNO Administration

Since the effect of GSNO on 35 HPs was transient, it was possible to evaluate stability/reproducibility of our detection and data processing system which was necessary to know in order to estimate the hysteresis of signaling pathways. In the following experiments, we tested how the detailed changes of the APW shape are reproducible in high time and pressure resolution. For better visual comparison, plots (A) and (AA) presenting systolic BP in the figures are the same. The plot of the augmentation index relative (e.g. Figure 2(a), (JJ)) was not possible to determine in cases when the highest point at APW was *c* and not *f* (Figure 1; note that point *f* representing maximal systolic BP can be different from the point *c* representing the 1st maximal BP).

The time-dependent changes of 35 HPs after 4 times consecutive administration of NO donor, 32 nmol kg^−1^ GSNO, are shown in Figure 2(a). The time-dependent changes of 35 HPs were well reproducible after the second and the third but less after the fourth GSNO administration. Some, but not all, HPs “followed” the time-dependent changes of systolic or diastolic BP.

In order to find “patterns” of the time-dependent parallel changes between HPs, their crossrelationships were studied. An example of the crossrelationships between the HPs and the systolic BP after the first, second, and third subsequent administrations of GSNO shows again that the patterns were reproducible (Figure 2(b)). It is noticeable that data between the first and the second administration are reproducible within ~1-2 mmHg and 1-2 ms, or e.g., the changes of time interval “DiN-AnN” were reproducible within the window of 6 ms (Figure 2(b), (FF)). The high sensitivity of the recorded system proved not only the clear detection of the anacrotic notch (Figure 1(a), *d*), but also the detection of the time and pressure fluctuation of diastolic BP between points a1 and a2, as it is seen from nonfiltered time-dependent HPs (Figure 1(b)).

Comparison of the crossrelationship between the HPs and systolic BP after the first and the fourth administration of GSNO shows that the patterns of the relationships of the fourth administration were not properly preserved or some of the relationships were “shifted” (Figure S1) indicating that reproducibility was lower than in the previous case (Figure 2(b)). It is supposed that the lower reproducibility was due to the time-dependent reduced efficiency of anesthetics.

The crossrelationship of HPs of the three GSNO administrations revealed a pattern for each HP. For example, in the systolic BP-HP crossrelationship, nonhysteresis (approximate linear) relationships were observed between systolic BP and (RL/D is an acronym for relative level/delay) the following: *dP*/*dt*_max_‐RL (E), *dP*/*dt*_*d*_‐RL (G), diastolic BP (J), pulse BP (K), *dP*/*dt*_min_ (M), *dP*/*dt*_*d*_ − *dP*/*dt*_max_ (Q), AnN (BB), AnN delay (DD), DiN (KK), DiN − AnN (OO), and (DiN − AnN)/*dP*/*dt*_max_ (QQ) (Figure 2(b)). It is supposed that the nonhysteresis relationship indicates that the signaling pathway(s) of HPs, responding to NO concentration, is directly connected; i.e., the NO signaling pathway(s) regulates directly both of the HPs.

Hysteresis loop relationships were observed between systolic BP and the following: heart rate (B), systolic area (C), *dP*/*dt*_max_ (D), *dP*/*dt*_*d*_ (F), *dP*/*dt*_*d*_ − *dP*/*dt*_max_ (H), diastolic area (L), *dP*/*dt*_min_ delay (O), *dP*/*dt*_*d*_ delay (P), Din − AnN (FF), (DiN − AnN)/*dP*/*dt*_max_ (HH), DiN delay (MM), or DiN-RD (NN). The hysteresis loop relationship indicates that the signaling pathway(s) of these HPs is delayed of each other or they are indirectly connected with systolic BP. Some pathways are faster than others, and/or “the later” pathways are responding to “the earlier” ones, causing the hysteresis delay. It is noticed that even in the case when the pattern of the third administration (green line) was shifted (e.g. Figure 2(b), (B), (H), (P)), the shape of the patterns remained similar. One can characterize the crossrelationship between the particular HP (taken from the 35 HPs) and other HPs and describe totally the 595 plots. Each of these crossrelationships reflects a specific connection between signaling pathways regulating the two particular HPs. Each plot of the two HPs is the pattern for the specific cardiovascular condition, and some of them may be unique for changes of NO concentration in the cardiovascular system. Figure 2(b) shows a 2-dimensional relationship only. The time dimension could be obtained if three-dimensional graphs are constructed. However, we found them too complex for visual presentation.

### 3.2. Nonhysteresis/Hysteresis Relationships between HPs during Transient Increased NO Bioavailability

**Figure 3:**
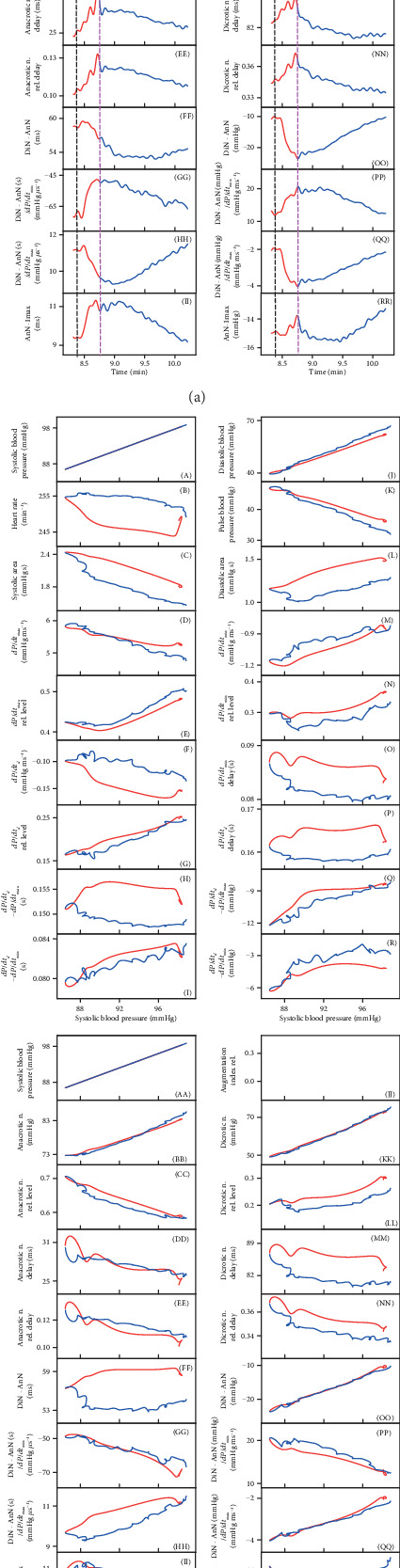
(a) Time-dependent changes of HPs of anesthetized rat after i.v. bolus administration of 32 nmol kg^−1^ GSNO (marked by black dash lines). The minimum value of systolic BP is marked by pink dash lines. The red line starts 3 s before GSNO administration. The red part of the curve corresponds to the decrease of systolic BP and the blue one to the increase of systolic BP. (b) Relationships of HPs to systolic BP after administrations of 32 nmol kg^−1^ GSNO. The red line represents the decrease of systolic BP from the control BP before GSNO administration to the lowest BP, and the blue line represents the increase of systolic BP from the lowest systolic BP to the control systolic BP ((A) or (AA) of (a)). The hysteresis was arbitrary defined as HP-systolic BP loop > 5 mmHg of systolic BP. (c) Nonhysteresis/hysteresis patterns of the relationships of HPs to systolic BP after administrations of 32 nmol kg^−1^ GSNO. Data were taken from (b) and S2. The total number of rats in which nonhysteresis (blue) or hysteresis (red) patterns were observed is *n* = 10. The hysteresis was arbitrarily defined as HP-systolic BP loop > 5 mmHg of systolic BP.

To look for details of patterns of crossrelationships between HPs and the connection between different pathways during transient increase of NO bioavailability, we better evaluated the time interval of decrease (red line) and increase (blue line) of systolic BP after GSNO administration.  Figure 3(a) shows an example of the time-dependent changes of 35 HPs. Some HPs did follow, but some did not, the decrease/increase of systolic BP, as shown, e.g., in plots (B), (F), (H), (L), (N), (O), (P), (R), (EE), (FF), (MM), or (RR).  Figure 3(b) shows an example of the relationship of 34 HPs to systolic BP after administration of GSNO. The relationship of HPs to systolic BP in the other nine rats is shown in Figures S2A-S2I. The data taken from Figure 3(b) and S2A-S2I from ten rats revealed nonhysteresis/hysteresis relationships, “direct” or “delayed-indirect” connections, between different pathways (Figure 3(c)).

The nonhysteresis or mostly nonhysteresis relationships (direct signaling pathways) were observed between systolic BP and the following: diastolic BP (J), pulse BP (K), *dP*/*dt*_min_ (M), AnN (BB), *dP*/*dt*_*d*_-RL (G), AnN delay (DD), AnN–1max (II), DiN (KK), *dP*/*dt*_max_ (D), *dP*/*dt*_max_-RL (E), *dP*/*dt*_*d*_ − *dP*/*dt*_max_ (Q), DiN − AnN (OO), (DiN − AnN)/*dP*/*dt*_min_ (PP), or (DiN − AnN)/*dP*/*dt*_max_ (QQ).

The pronounced hysteresis and/or approximately wedge-shaped hysteresis (delayed-indirect signaling pathways) relationships were observed between systolic BP and the following: systolic area (C), *dP*/*dt*_*d*_ (F), diastolic area (L), *dP*/*dt*_min_-RL (N), *dP*/*dt*_min_ delay (O), *dP*/*dt*_*d*_ − *dP*/*dt*_min_ (R), DiN − AnN (FF), (DiN − AnN)/*dP*/*dt*_max_ (HH), DiN-RL (LL), DiN delay (MM), heart rate (B), *dP*/*dt*_*d*_ − *dP*/*dt*_max_ (H), *dP*/*dt*_*d*_ delay (P), or AnN–1max (RR). The nonhysteresis/hysteresis responses were not pronounced in the rest of the HPs (Figure 3(c)). Using the same procedure for each couple of 35 HPs, one can describe totally the 595 direct/indirect signaling pathways.

### 3.3. Rates of Time-Dependent Changes of HPs after Transient Increase of NO Bioavailability

**Figure 4:**
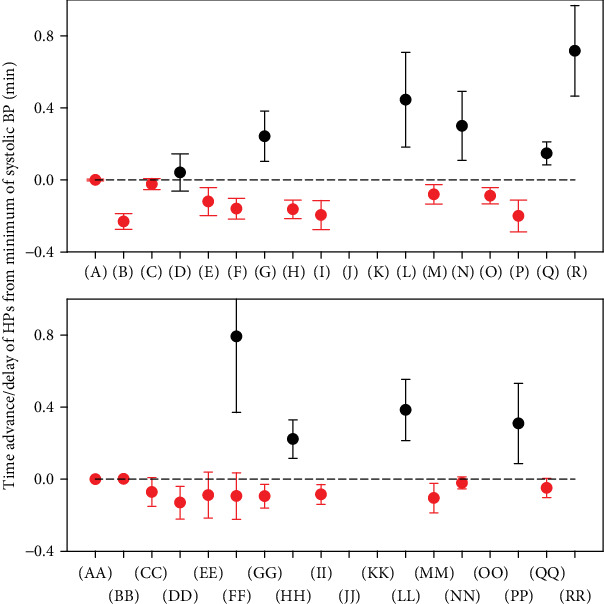
Comparison of time advance/delay of the responses of HPs with comparison to minimum of systolic BP (0 min, Figure 3(a)) after i.v. administration of GSNO (means ± SE; *n* = 10). The red circle symbol represents the average advance response and the black one the average delay response.

The time-dependent changes of HPs after GSNO administration (Figure 3(a)) allowed us to estimate and compare rates of the changes of different HPs to the transient decrease of systolic BP. To compare the rates of the responses of HPs with changes of systolic BP after GSNO administration, the time differences between local minimum or maximum of the time-dependent values of HPs and the control minimum of systolic BP (pink line in Figure 3(a)) were manually evaluated. The rate of HP changes, depicted as plots (B), (E), (F), (H), (I), (M), (O), (P), (DD), (EE), (FF), (GG), (II), and (MM), was faster (at least part of them) than the rate of changes of systolic BP. The changes of HPs, depicted as plots (D), (G), (L), (N), (Q), (R), (FF), (HH), (LL), and (PP), were slower than the changes of systolic BP. The rates of other HPs were not clear (Figure 4). The different rates may reflect different “speeds” of signaling pathways responsible for the changes of the related HPs.

### 3.4. Effect of Increase of NO Bioavailability on HPs

**Figure 5:**
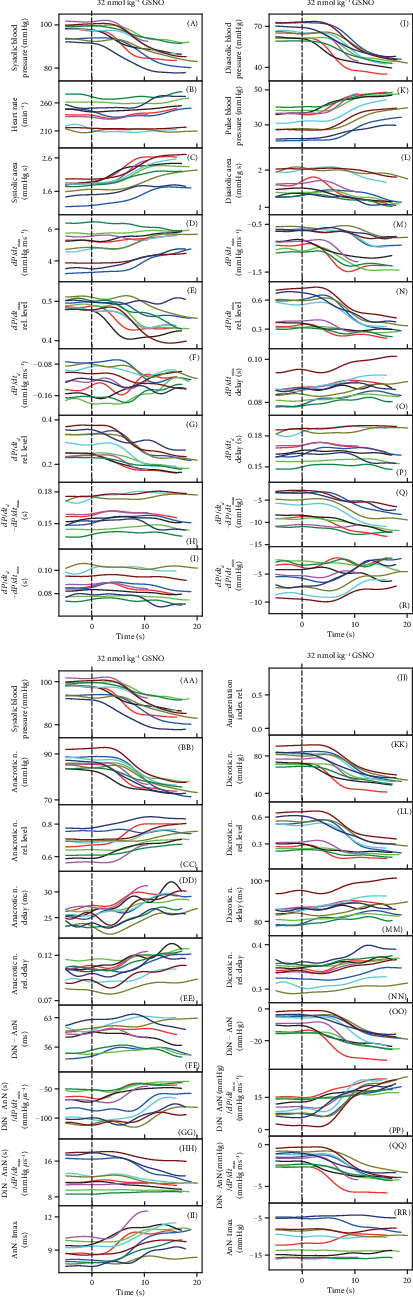
Time-dependent changes of HPs of anesthetized rat after i.v. bolus administration of 32 nmol kg^−1^ GSNO (marked by dash line). The decreasing part of the systolic BP was evaluated only; *n* = 10. Each color represents an individual rat.

**Figure 6:**
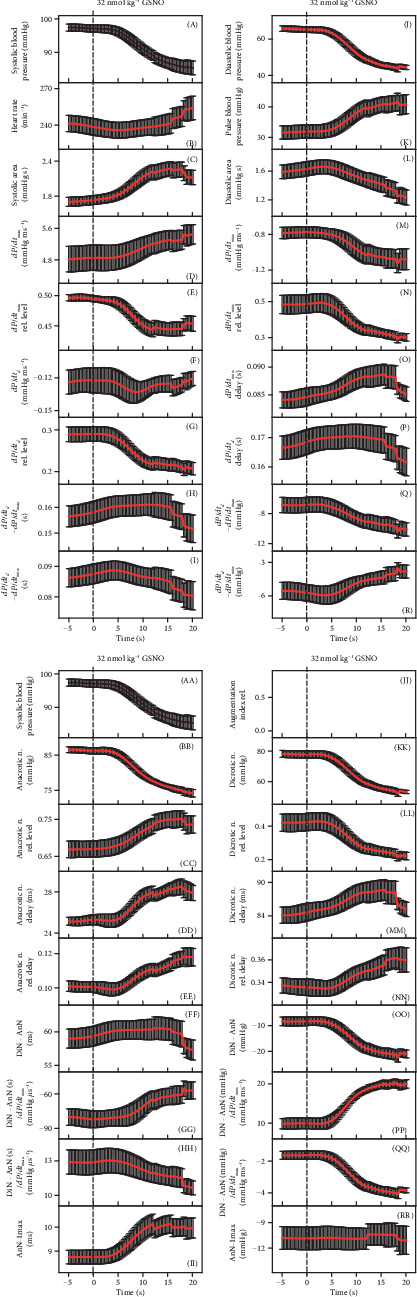
Time-dependent changes of HPs of anesthetized rat after i.v. bolus administration of 32 nmol kg^−1^ GSNO (marked by dash line). The decreasing part of systolic BP was evaluated only. The data are means (SE) of values from Figure 5 (*n* = 10).

Since the decrease of systolic BP was observed during GSNO administration, it is supposed to reflect the increase of NO bioavailability. The time-dependent changes of HPs during the decrease of systolic BP are shown in Figure 5. Each color in the figure represents individual rats. Systolic BP of control measurements was mostly (7/10) in the range 97-102 mmHg. However, most of the other control HPs (before GSNO administration) had apparently greater variability, e.g., see plots (B), (D), (F), (H), (I), (L), (N), (O), (P), (Q), (R), (CC), (DD), (EE), (FF), (GG), (HH), (LL), (NN), or (RR) (Figure 5). After GSNO administration, some of the HP values were “scattered” more, e.g., systolic BP (A) or systolic area (C), and some of them less, e.g., *dP*/*dt*_max_ (D), *dP*/*dt*_min_-RL (N), or dicrotic notch relative level (LL), indicating a complex effect of NO on the cardiovascular system (Figure 5).  Figure 6 shows the average time-dependent changes of 35 HPs observed on 10 independent rats during increase of NO bioavailability resulting from GSNO administration. The time-dependent changes were observed in most of the HPs.

### 3.5. Comparison of Increase with Prolonged Decrease of NO Bioavailability on 35 HPs

In our previous study, we evaluated time-dependent changes of 35 HPs in the condition of prolonged decrease of NO bioavailability caused by administration of NOS inhibitor, N(*ω*)-nitro-L-arginine methyl ester (L-NAME) (see Figure 3 in [20]). We compared those data with the data obtained under the conditions of increased NO bioavailability by GSNO (Figure 6). The comparison of the effects of time-dependent increase (by GSNO) and decrease (by L-NAME) of NO bioavailability on HPs revealed that only 17 out of 35 HPs have an opposite clear trend indicating a possible direct relation to NO bioavailability (Table 1). Interestingly, 10 HPs have the same time-dependent trend during increase or decrease of NO bioavailability, which may indicate that they reflect the cardiovascular system in an optimal NO bioavailability condition. For example, pulse BP, as an important parameter of the cardiovascular system reflecting arterial stiffness, increased during both increasing and decreasing NO bioavailability. Effect on the remaining 8 HPs was even more complex, showing an unclear or noncorrelated biphasic time-dependent response during increase and decrease of NO bioavailability.

### 3.6. Effect of Transient Increase in NO Bioavailability on Distinct Fluctuation of Diastolic BP

**Figure 7:**
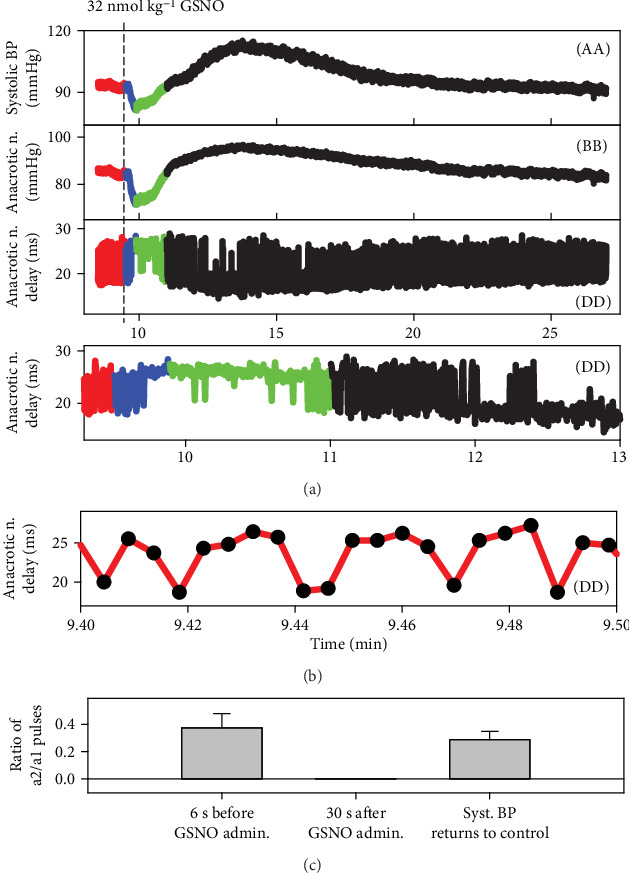
Time-dependent effect of 32 nmol kg^−1^ GSNO on three HPs. The fluctuations of anacrotic notch delay (dd) reflect the time interval fluctuation between the a1 and a2 points (Figure 1). The higher value of anacrotic notch delay in ms (a, b) indicates that the diastolic BP at point a1 was lower than that at point a2. The fluctuation between the a1 and a2 points in pulses (black circles) recorded for 6 s before the GSNO administration (b). Comparison of the ratio of a2/a1 pulses during 6 s: 6 s before, 30 s after the GSNO administration, and at the time when systolic BP returned to the control value (c); means ± SE, *n* = 10.

Administration of GSNO influenced also a minor fluctuation of diastolic BP. The high sensitivity of the recording system detected the distinct diastolic BP fluctuation between two points (Figure 1, points a1 and a2), which is clearly seen from the measurement of time interval between anacrotic notch (*d*) and diastolic BP (a1 or a2), and this time interval (*d* − a1) or (*d* − a2) can be depicted as the plot of anacrotic notch delay (Figure 7(a), (DD)). The higher value of anacrotic notch delay in ms (Figure 7(b)) indicates that diastolic BP at point a1 was lower than at point a2. The time dependence of the anacrotic notch delay more or less reflected the time-dependent changes of systolic BP and the position of the anacrotic notch in mmHg (Figure 7(a)). The comparison of the fluctuation between the a1 and a2 values at single pulses, the ratio of a2/a1 pulses, showed that 30 s after the GSNO administration, when systolic BP decreased, the a2/a1 ratio decreased to zero and returned back when systolic BP returned to the control values—before GSNO administration (Figure 7(c)).

## 4. Discussion

Our work is based on the hypothesis that it is possible to characterize the cardiovascular system in pathophysiological conditions just from the shape of APW [20]. From the data, it is evident that the presented approach detects subtle changes of APW with sufficient reproducibility under equal experimental conditions. The NO donor GSNO was i.v. administered continuously for 15 s, during which systolic BP gradually decreased for ~18 s, and later, BP increased. This is in agreement with fast inactivation of NO in plasma by binding to oxyhemoglobin with subsequent conversion to methemoglobin and nitrate [4, 25] and with reports that nitrovasodilators produce characteristic changes in the shape of a rabbit peripheral pulse wave specific to NO [17, 26].

The origin of the short (width~10 ms) and deep (~5-15 mmHg) anacrotic notch (point *d* in Figure 1(a)) is not yet fully understand. It may result from a sudden decrease in the rate of acceleration of pressure, sudden and short increase of blood volume of the arterial tree, and/or reflection waves from the arterial tree. We do not know what the minor time/pressure fluctuation of diastolic BP (Figures 1(a) and 1(b), point a—a1 or a2, and Figure 7) reflects. But the observations that point *a* fluctuated between two distinct positions and that it was influenced by NO bioavailability and by decrease/increase of BP indicate that it might be (patho-) physiologically important (Figure 7).

It is suggested that the nonhysteresis of the crossrelationship between two particular HPs revealed a direct connection between the signaling pathways regulating these two HPs, whereas hysteresis revealed that the signaling pathways are indirectly connected or they are time delayed of each other (Figures 2(b), 3(b), and 3(c)). The HPs related to the NO/cGMP-mediated relaxation of periphery and coronary blood vessels, e.g., the diastolic BP, pulse BP, and *dP*/*dt*_min_, were found to be in a nonhysteresis relationship to BP. Some of the HPs in a hysteresis relationship with BP, e.g., heart rate, *dP*/*dt*_max_, systolic area, and dicrotic notch delay, reflected the reflex response of the heart to BP changes. Each plot of the crossrelationship between two HPs is the pattern for the particular cardiovascular condition, and some of them may be unique for changes of NO concentration in the cardiovascular system. Similarly, from the comparison of the rates of decrease/increase of HPs (Figure 4), it is suggested that the rates of HPs, which followed the rate of systolic BP change, reflect the direct connection between signaling pathways and the rates of HPs, and those, which are different to the systolic BP, reflect the signaling pathways indirectly connected or they are time delayed of each other.

The observation that in spite of stabilization of baseline systolic BP to ~100 mmHg (7/10), other baseline HPs are not in the same compact range (Figure 5) indicates that using systolic BP alone as a standard *in vivo* experimental condition in animal models should be taken with caution. There are two possible reasons for the greater variability of control HP values. At the ~100 mmHg of systolic BP, other HPs are not stabilized because they reflect subtle details of cardiovascular conditions resulting from individual physiological conditions or from the different effect of anesthesia on a particular rat. Since anesthesia influences HPs [27], it is supposed that a different level of anesthesia may contribute to the data variability differently.

It was expected that the trend of the time-dependent changes of most of the HPs should be opposite during increase (by GSNO) and decrease (by L-NAME) of NO bioavailability. However, only 17 out of 35 HPs had a clear opposite trend indicating that these HPs reflect a possible direct relation of the parameters to NO bioavailability. For instance, it was suggested that the changes in the relative level of the dicrotic notch are specific for NO signaling [17, 26]. We found that the relative level of the anacrotic and the dicrotic notch corresponded with the NO bioavailability positively and negatively, respectively, and these changes were in a nonhysteresis relationship with BP. Remarkably, 10 HPs had the same time-dependent trend during the increase or decrease of NO bioavailability indicating that they describe how altered NO bioavailability “detunes” the cardiovascular system in a similar direction. This may indicate that these HPs reflect optimal NO bioavailability. Effect on the remaining 8 HPs was even more complex, showing an unclear or biphasic time-dependent response during the increase/decrease of NO bioavailability (Table 1). The data reveal a complex response of the cardiovascular system to NO bioavailability. However, the actual meaning of most HPs and their crossrelationships to particular signaling pathways is unknown, remaining a challenge for the future study.

## 5. Conclusion

Our work provides a new insight of exploring APW to characterize novel details of the cardiovascular system, in particular, conditions, and presents numerous original data characterizing APW by new HPs and by their crossrelationships. The time-dependent changes of 35 HPs were simultaneously evaluated and revealed details of the APW shape at high time and BP resolutions, which enabled us to detect, e.g., the anacrotic notch, the minor time/pressure fluctuation of diastolic BP, and to define several new HPs and present simultaneously their time-dependent changes and crossrelations. Since effects of NO were transient, we showed the different rates and nonhysteresis/hysteresis time-dependent changes of 35 HPs, and from their crossrelationships to systolic BP, the “patterns” and direct/indirect signaling pathways were suggested.

The observed time-dependent changes of 35 HPs and their crossrelationships can serve as patterns for conditions of transient increase/decrease of NO. However, to determine which of the patterns are “unique” for increase/decrease of NO, more studies are needed to define the cardiovascular system in different particular conditions. The specific patterns may reveal details of function, connection, and/or cooperation of different signaling pathways in the cardiovascular system. The “unique patterns” for NO could be determined only after knowing patterns for different pathophysiological cardiovascular conditions, e.g., when specific membrane channels are blocked or activated by selective drugs and when specific receptors or particular signaling pathways are activated and inhibited. From a clinical perspective, this approach may be used in animal models and in humans to create a data bank of patterns of crossrelationships of HPs for different cardiovascular conditions. By comparison of unknown patterns of studied APW with the data bank patterns, it would be possible to determine cardiovascular conditions of the studied subject from the recorded arterial BP. It can help to find the molecular mechanism of particular (patho-) physiological conditions or drug action and may have predictive or diagnostic value.

## Figures and Tables

**Table 1:** Comparison of time-dependent effects of increased (32 nmol kg^−1^ GSNO; Figure 6) and decreased (25 mg kg^−1^ L-NAME; data from Figure 3 in [20]) NO concentrations on HPs.

Description	GSNO	L-NAME
(A) Systolic blood pressure (in mmHg)	↓	↑
(B) Heart rate (in min^−1^)	~	↓
(C) Systolic area (in mmHg s)	↑	↑
(D) *dP*/*dt*_max_ (in mmHg ms^−1^)	↑	↓
(E) *dP*/*dt*_max_ relative level	↓	↓
(F) *dP*/*dt*_*d*_ (in mmHg ms^−1^)	↓↑	↑↓
(G) *dP*/*dt*_*d*_ relative level	↓	↑↓
(H) *dP*/*dt*_*d*_ − *dP*/*dt*_max_ (in s)	↑	↑
(I) *dP*/*dt*_*d*_ − *dP*/*dt*_min_ (in s)	↑↓	↑
(J) Diastolic blood pressure (in mmHg)	↓	↑
(K) Pulse blood pressure (in mmHg)	↑	↑
(L) Diastolic area (in mmHg s)	↓	↑
(M) *dP*/*dt*_min_ (in mmHg ms^−1^)	↓	↓
(N) *dP*/*dt*_min_ relative level	↓	↑↓
(O) *dP*/*dt*_min_ delay (in s)	↑	↑
(P) *dP*/*dt*_*d*_ delay (in s)	↑	↑
(Q) *dP*/*dt*_*d*_ − *dP*/*dt*_max_ (in mmHg)	↓	↑
(R) *dP*/*dt*_*d*_ − *dP*/*dt*_min_ (in mmHg)	↑	↓
(AA) Systolic blood pressure (in mmHg)	↓	↑
(BB) Anacrotic notch (in mmHg)	↓	↑
(CC) Anacrotic notch relative level	↑	↓
(DD) Anacrotic notch delay (in ms)	↑	↓
(EE) Anacrotic notch relative delay	↑	↓
(FF) Dicrotic notch (DiN) − Anacrotic notch (AnN) (in s)	↑	↑
(GG) (DiN − AnN)/*dP*/*dt*_min_ (in s/mmHg *μ*s^−1^)	↑	↑
(HH) (DiN − AnN/*dP*/*dt*_max_ (in s/mmHg *μ*s^−1^)	↓	↑
(II) AnN − 1max (in ms)	↑	↓
(JJ) Augmentation index relative	~	↑
(KK) Dicrotic notch (in mmHg)	↓	↑
(LL) Dicrotic notch relative level	↓	↑↓
(MM) Dicrotic notch delay (in ms)	↑	↑
(NN) Dicrotic notch relative delay	↑	↓
(OO) DiN − AnN (in mmHg)	↓	↑
(PP) (DiN − AnN)/*dP*/*dt*_min_ (in mmHg/mmHg ms^−1^)	↑	↓
(QQ) (DiN − AnN)/*dP*/*dt*_max_ (in mmHg/mmHg ms^−1^)	↓	↑
(RR) AnN − 1max (in mmHg)	~	↑

## Data Availability

All findings and conclusions are based on the presented figures in the main text or in the supplementary information. Original source files can be sent from the corresponding author, Dr. Karol Ondrias, upon request.

## Supplementary Materials

Supplementary Materials Figure S1: relationships of HPs to systolic BP after the first and the fourth administration of 32 nmol kg^−1^ GSNO. The data and colors were taken from Figure 2(a). Figure S2A: relationships of HPs to systolic BP after administration of 32 nmol kg^−1^ GSNO. The red line represents the decrease of the systolic BP from the control BP to the lowest BP, and the blue line represents the increase of the systolic BP from the lowest systolic BP to the control systolic BP (Figure 3(a), (A) or 3(a), (AA)). Figure S2B: relationships of HPs to systolic BP after administration of 32 nmol kg^−1^ GSNO. The red line represents the decrease of the systolic BP from the control BP to the lowest BP, and the blue line represents the increase of the systolic BP from the lowest systolic BP to the control systolic BP (Figure 3(a), (A) or 3(a), (AA)). Figure S2C: relationships of HPs to systolic BP after administration of 32 nmol kg^−1^ GSNO. The red line represents the decrease of the systolic BP from the control BP to the lowest BP, and the blue line represents the increase of the systolic BP from the lowest systolic BP to the control systolic BP (Figure 3(a), (A) or 3(a), (AA)). Figure S2D: relationships of HPs to systolic BP after administration of 32 nmol kg^−1^ GSNO. The red line represents the decrease of the systolic BP from the control BP to the lowest BP, and the blue line represents the increase of the systolic BP from the lowest systolic BP to the control systolic BP (Figure 3(a), (A) or 3(a), (AA)). Figure S2E: relationships of HPs to systolic BP after administration of 32 nmol kg^−1^ GSNO. The red line represents the decrease of the systolic BP from the control BP to the lowest BP, and the blue line represents the increase of the systolic BP from the lowest systolic BP to the control systolic BP (Figure 3(a), (A) or 3(a), (AA)). Figure S2F: relationships of HPs to systolic BP after administration of 32 nmol kg^−1^ GSNO. The red line represents the decrease of the systolic BP from the control BP to the lowest BP, and the blue line represents the increase of the systolic BP from the lowest systolic BP to the control systolic BP (Figure 3(a), (A) or 3(a), (AA)). Figure S2G: relationships of HPs to systolic BP after administration of 32 nmol kg^−1^ GSNO. The red line represents the decrease of the systolic BP from the control BP to the lowest BP, and the blue line represents the increase of the systolic BP from the lowest systolic BP to the control systolic BP (Figure 3(a), (A) or 3(a), (AA)). Figure S2H: relationships of HPs to systolic BP after administration of 32 nmol kg^−1^ GSNO. The red line represents the decrease of the systolic BP from the control BP to the lowest BP, and the blue line represents the increase of the systolic BP from the lowest systolic BP to the control systolic BP (Figure 3(a), (A) or 3(a), (AA)). Figure S2I: relationships of HPs to systolic BP after administration of 32 nmol kg^−1^ GSNO. The red line represents the decrease of the systolic BP from the control BP to the lowest BP, and the blue line represents the increase of the systolic BP from the lowest systolic BP to the control systolic BP (Figure 3(a), (A) or 3(a), (AA)).
