# Supplementary material for: Patterns and Direct/Indirect Signaling Pathways in Cardiovascular System in the Condition of Transient Increase of NO
Source: Biomed Res Int. 2020 May 27;2020:6578213. doi: 10.1155/2020/6578213 (PMC7275240; doi:10.1155/2020/6578213)
Supplement: Supplementary Materials — Figure S1: relationships of HPs to systolic BP after the first and the fourth administration of 32 nmol kg−1 GSNO. The data and colors were taken from Figure 2(a). Figure S2A: relationships of HPs to systolic BP after administration of 32 nmol kg−1 GSNO. The red line represents the decrease of the systolic BP from the control BP to the lowest BP, and the blue line represents the increase of the systolic BP from the lowest systolic BP to the control systolic BP (Figure 3(a), (A) or 3(a), (AA)). Figure S2B: relationships of HPs to systolic BP after administration of 32 nmol kg−1 GSNO. The red line represents the decrease of the systolic BP from the control BP to the lowest BP, and the blue line represents the increase of the systolic BP from the lowest systolic BP to the control systolic BP (Figure 3(a), (A) or 3(a), (AA)). Figure S2C: relationships of HPs to systolic BP after administration of 32 nmol kg−1 GSNO. The red line represents the decrease of the systolic BP from the control BP to the lowest BP, and the blue line represents the increase of the systolic BP from the lowest systolic BP to the control systolic BP (Figure 3(a), (A) or 3(a), (AA)). Figure S2D: relationships of HPs to systolic BP after administration of 32 nmol kg−1 GSNO. The red line represents the decrease of the systolic BP from the control BP to the lowest BP, and the blue line represents the increase of the systolic BP from the lowest systolic BP to the control systolic BP (Figure 3(a), (A) or 3(a), (AA)). Figure S2E: relationships of HPs to systolic BP after administration of 32 nmol kg−1 GSNO. The red line represents the decrease of the systolic BP from the control BP to the lowest BP, and the blue line represents the increase of the systolic BP from the lowest systolic BP to the control systolic BP (Figure 3(a), (A) or 3(a), (AA)). Figure S2F: relationships of HPs to systolic BP after administration of 32 nmol kg−1 GSNO. The red line represents the decrease of the systolic BP from the contr [file 6578213.f1.pdf]

BioMed Research International

Supplementary Data

to

Patterns and direct/indirect signaling pathways in cardiovascular system in the condition of transient increase of NO

Anton Misak,[1] Lucia Kurakova,[1,2] Andrea Berenyiova,[3] Lenka Tomasova,[1] Marian Grman,[1] Sona Cacanyiova,[3] Karol Ondrias[1]

[1]Institute of Clinical and Translational Research, Biomedical Research Center, Slovak Academy of Sciences, Dubravska cesta 9, 845 05 Bratislava, Slovakia

[2]Department of Pharmacology and Toxicology, Faculty of Pharmacy, Comenius University, Odbojarov 10, 832 32 Bratislava, Slovakia

[3]Institute of Normal and Pathological Physiology, Centre of Experimental Medicine, Slovak Academy of Sciences, Dubravska cesta 9, 841 04 Bratislava, Slovakia

Correspondence should be addressed to Karol Ondrias, karol.ondrias@savba.sk

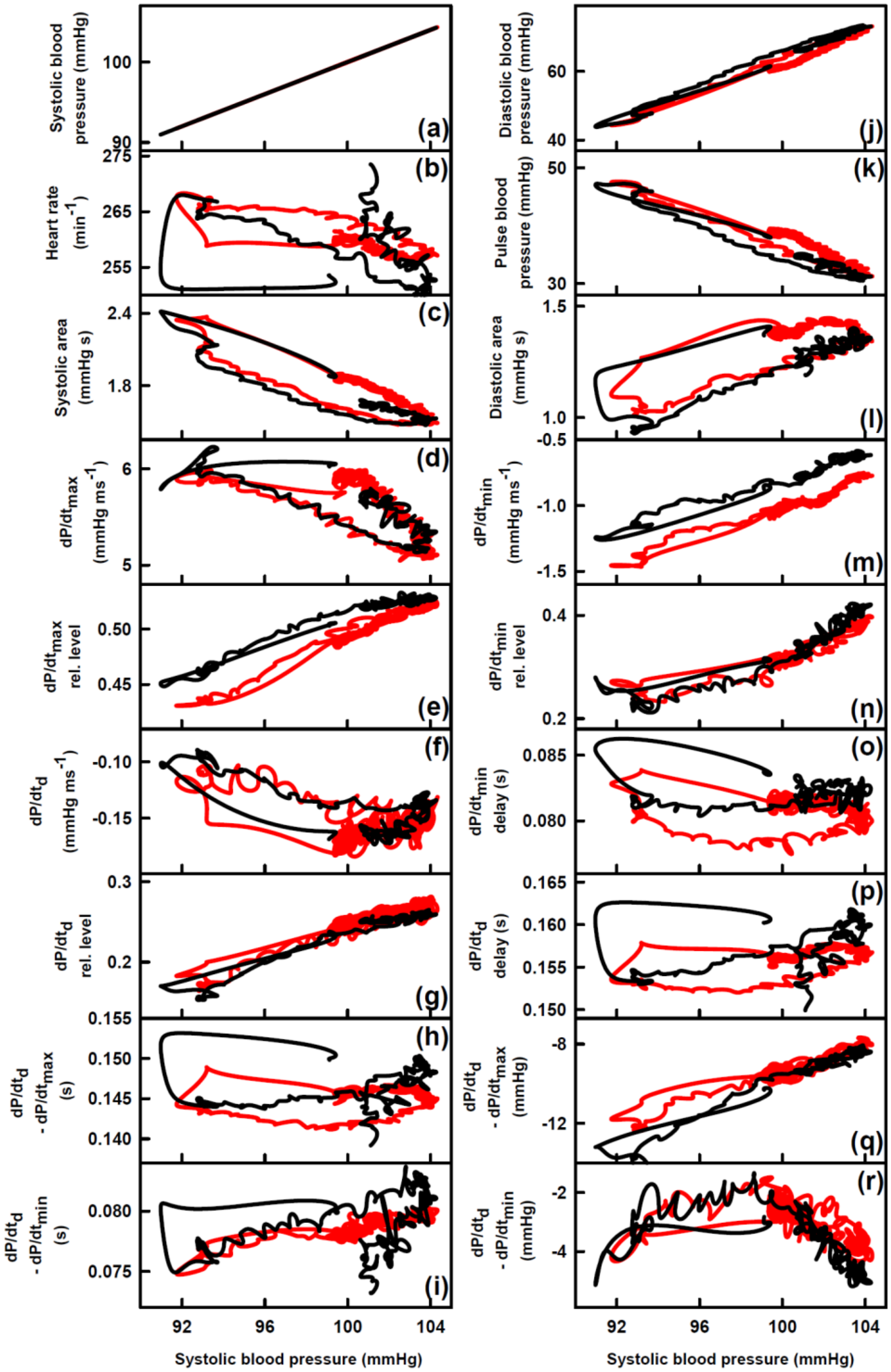
Systolic blood pressure (mmHg)
Heart rate (min-1)
Systolic area (mmHg s)
dP/dtmax (mmHg ms-1)
dP/dtmax rel. level
dP/dtd (mmHg ms-1)
dP/dtd rel. level
dP/dtd - dP/dtmax (s)
dP/dtd - dP/dtmin (s)
Diastolic blood pressure (mmHg)
Pulse blood pressure (mmHg)
Diastolic area (mmHg s)
dP/dtmin (mmHg ms-1)
dP/dtmin rel. level
dP/dtmin delay (s)
dP/dtd delay (s)
dP/dtd - dP/dtmax (mmHg)
dP/dtd - dP/dtmin (mmHg)
(a)
(b)
(c)
(d)
(e)
(f)
(g)
(h)
(i)
(j)
(k)
(l)
(m)
(n)
(o)
(p)
(q)
(r)
Systolic blood pressure (mmHg)
Systolic blood pressure (mmHg)

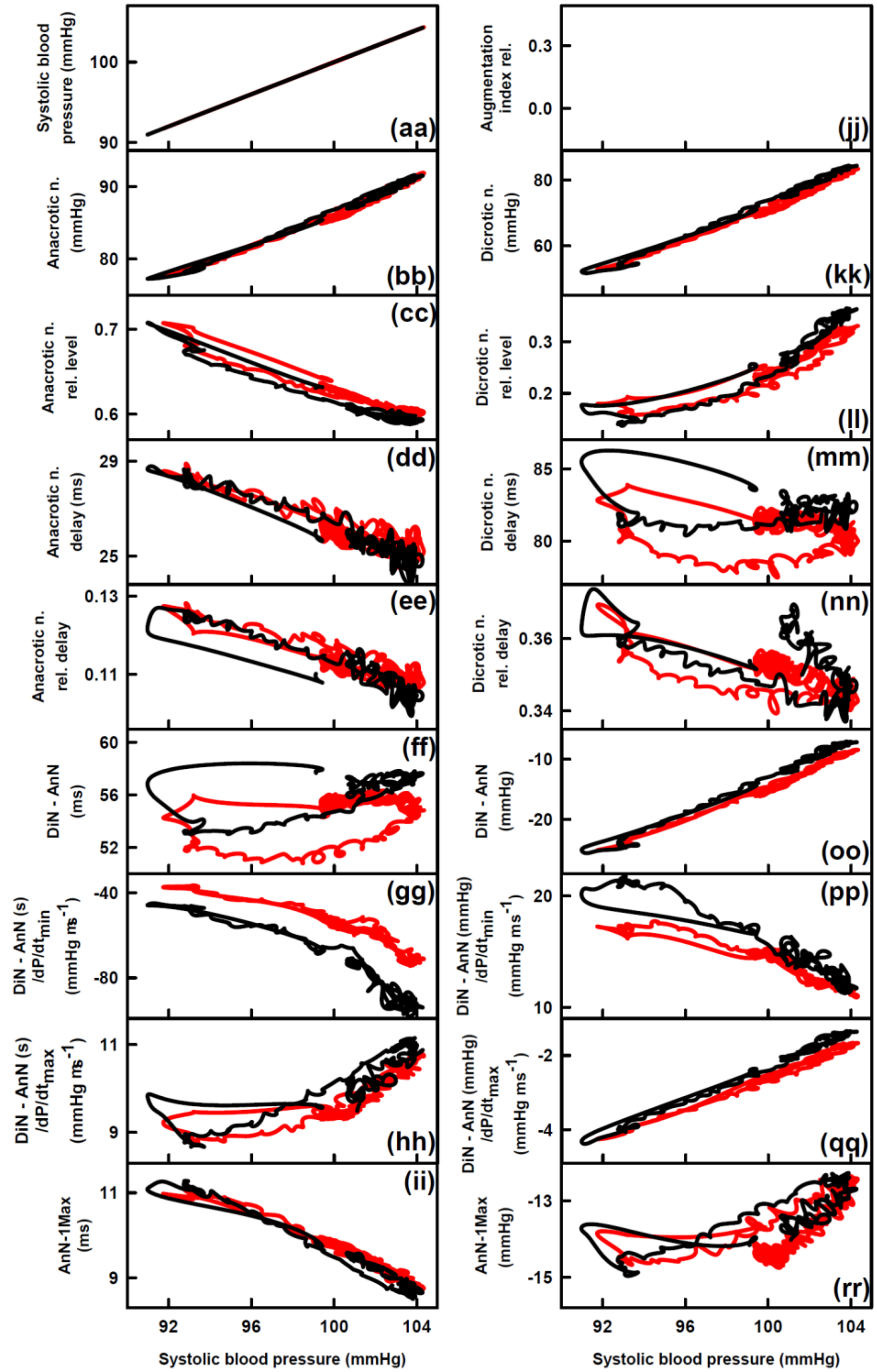

Figure S1 Relationships of HPs to systolic BP after the first and the fourth administration of 32 nmol kg^{-1} GSNO. The data and colours were taken from Figure 2A.

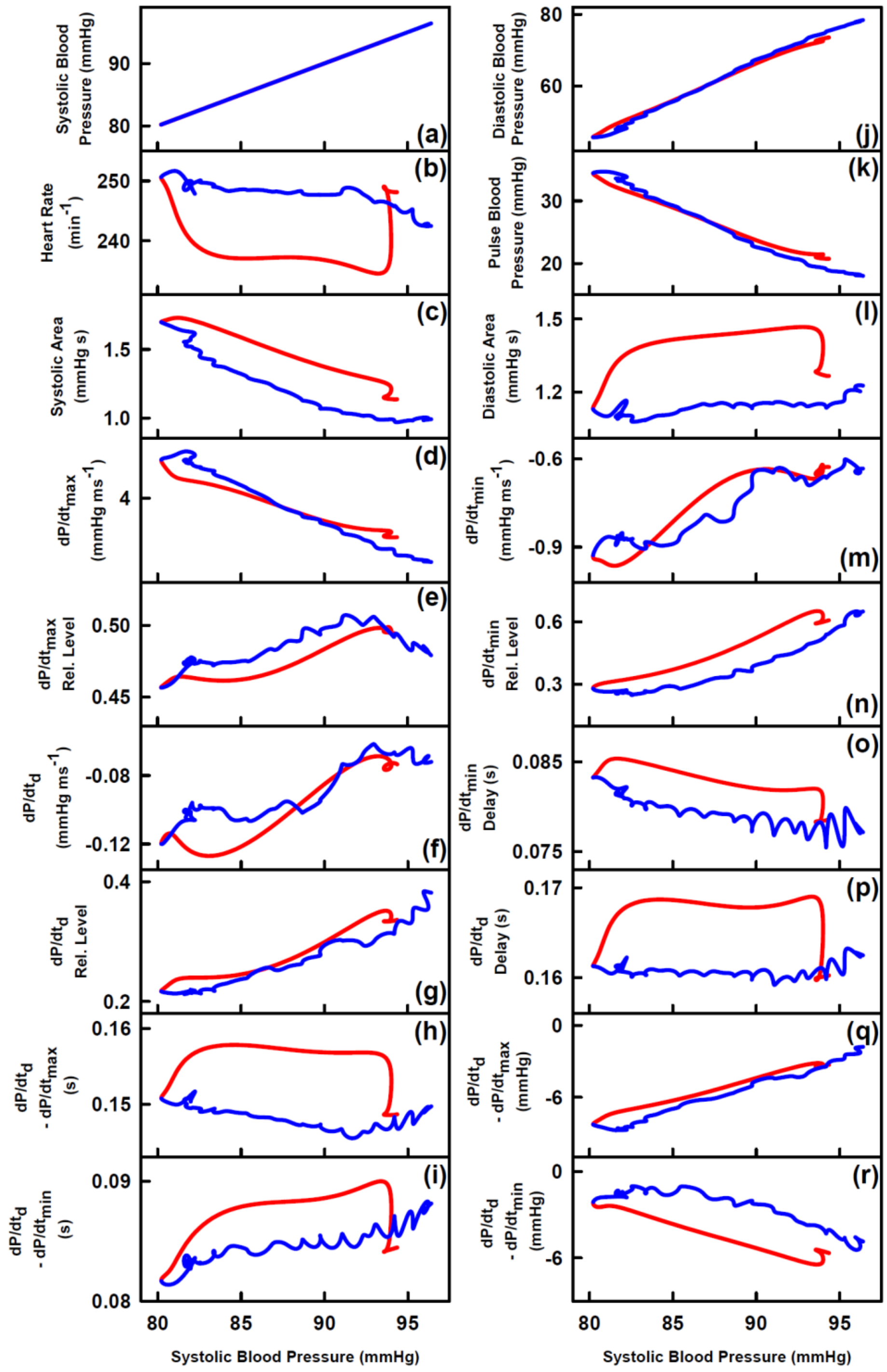
Systolic Blood Pressure (mmHg)
Heart Rate (min-1)
Systolic Area (mmHg s)
dP/dtmax (mmHg ms-1)
dP/dtmax Rel. Level
dP/dtd (mmHg ms-1)
dP/dtd Rel. Level
dP/dtd - dP/dtmax (s)
dP/dtd - dP/dtmin (s)
Diastolic Blood Pressure (mmHg)
Pulse Blood Pressure (mmHg)
Diastolic Area (mmHg s)
dP/dtmin (mmHg ms-1)
dP/dtmin Rel. Level
dP/dtmin Delay (s)
dP/dtd Delay (s)
dP/dtd - dP/dtmax (mmHg)
dP/dtd - dP/dtmin (mmHg)
(a) (b) (c) (d) (e) (f) (g) (h) (i) (j) (k) (l) (m) (n) (o) (p) (q) (r)
Systolic Blood Pressure (mmHg)
Systolic Blood Pressure (mmHg)

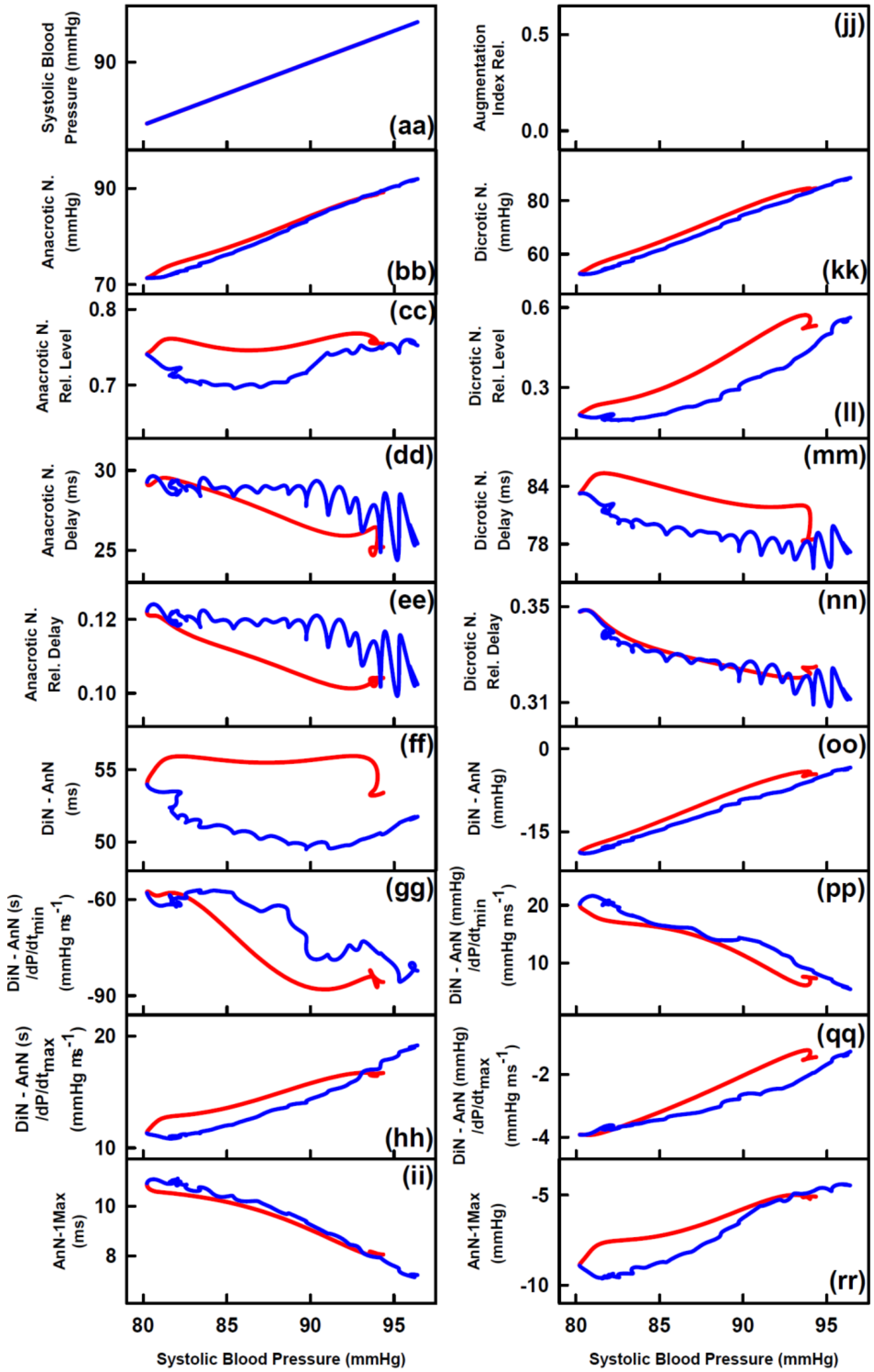

Figure S2A Relationships of HPs to systolic BP after administrations of 32 nmol kg^{-1} GSNO. The red line represents the decrease of the systolic BP from the control BP to the lowest BP and blue line represents the increase of the systolic BP from the lowest systolic BP to the control systolic BP (Figure 3A(a) or 3A(aa)).

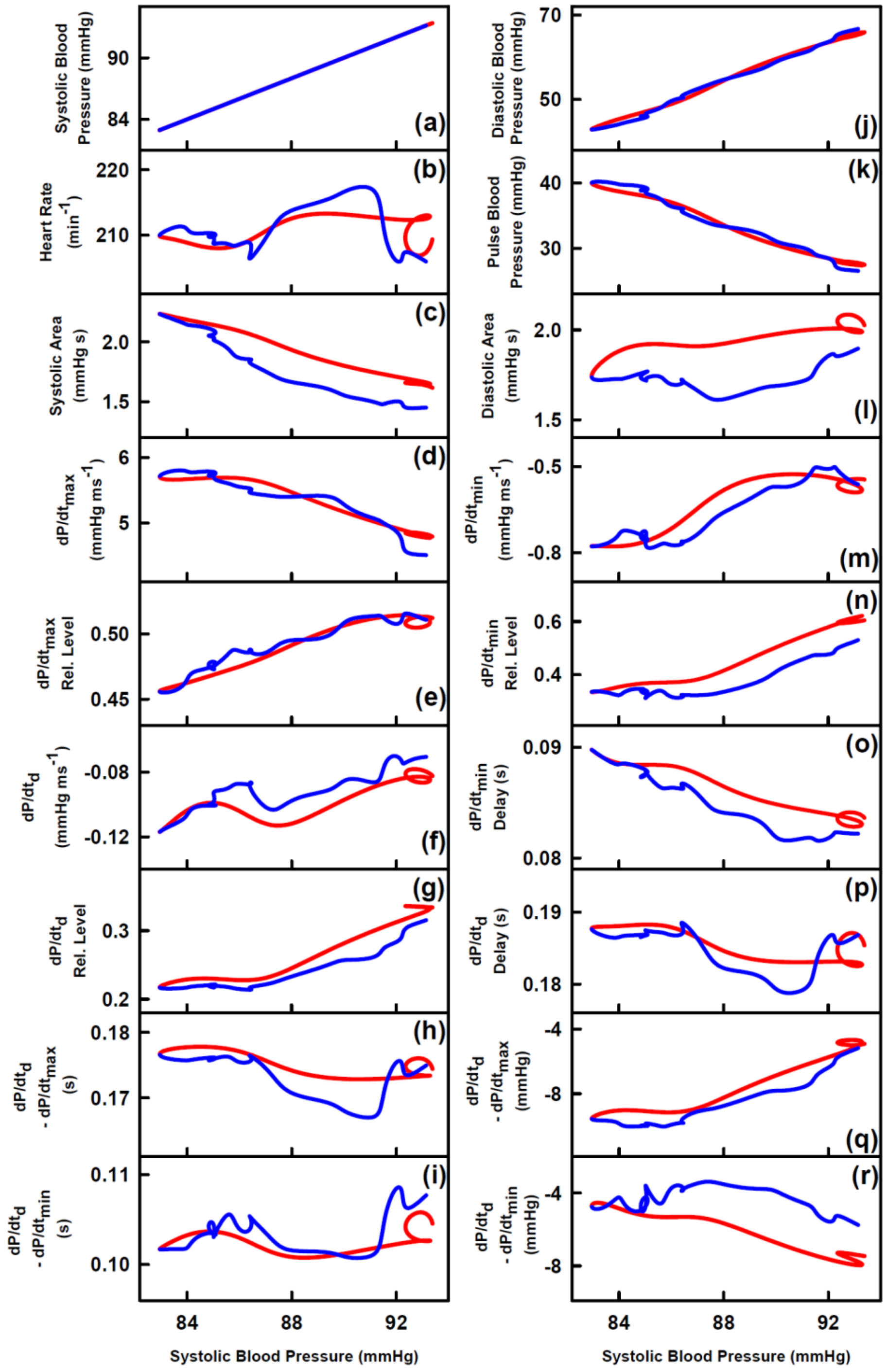

Systolic Blood Pressure (mmHg)
Heart Rate (min-1)
Systolic Area (mmHg s)
dP/dtmax (mmHg ms-1)
dP/dtmax Rel. Level
dP/dtd (mmHg ms-1)
dP/dtd Rel. Level
dP/dtd - dP/dtmax (s)
dP/dtd - dP/dtmin (s)
Diastolic Blood Pressure (mmHg)
Pulse Blood Pressure (mmHg)
Diastolic Area (mmHg s)
dP/dtmin (mmHg ms-1)
dP/dtmin Rel. Level
dP/dtmin Delay (s)
dP/dtd Delay (s)
dP/dtd - dP/dtmax (mmHg)
dP/dtd - dP/dtmin (mmHg)
(a) (b) (c) (d) (e) (f) (g) (h) (i)
(j) (k) (l) (m) (n) (o) (p) (q) (r)
Systolic Blood Pressure (mmHg)

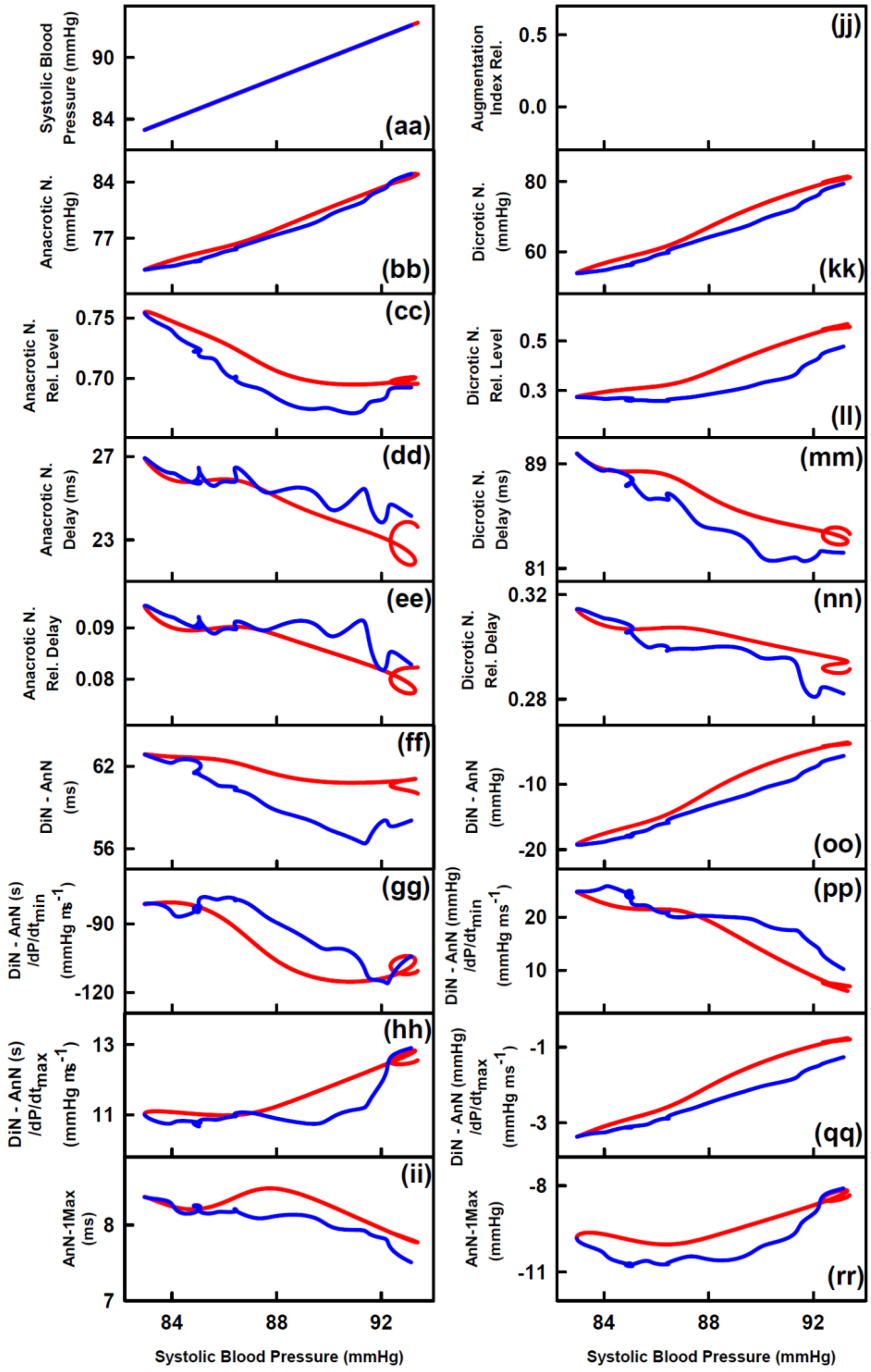

Figure S2B Relationships of HPs to systolic BP after administrations of 32 nmol kg^{-1} GSNO. The red line represents the decrease of the systolic BP from the control BP to the lowest BP and blue line represents the increase of the systolic BP from the lowest systolic BP to the control systolic BP (Figure 3A(a) or 3A(aa)).

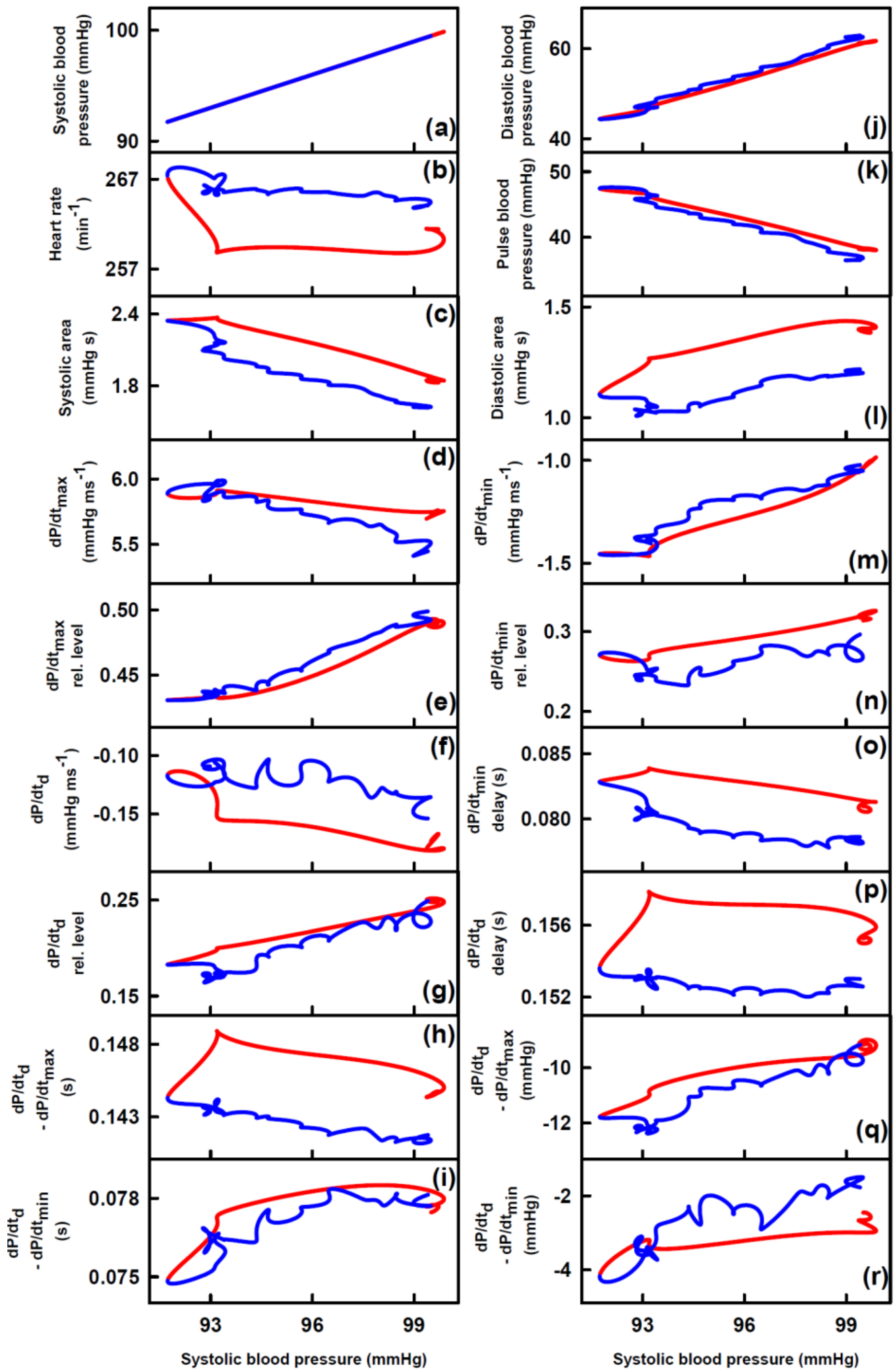

Systolic blood pressure (mmHg)
100
90
(a)
Heart rate (min-1)
267
257
(b)
Systolic area (mmHg s)
2.4
1.8
(c)
dP/dtmax (mmHg ms-1)
6.0
5.5
(d)
dP/dtmax rel. level
0.50
0.45
(e)
dP/dtd (mmHg ms-1)
-0.10
-0.15
(f)
dP/dtd rel. level
0.25
0.15
(g)
dP/dtd - dP/dtmax (s)
0.148
0.143
(h)
dP/dtd - dP/dtmin (s)
0.078
0.075
(i)
93
96
99
Systolic blood pressure (mmHg)
Diastolic blood pressure (mmHg)
60
40
(j)
Pulse blood pressure (mmHg)
50
40
(k)
Diastolic area (mmHg s)
1.5
1.0
(l)
dP/dtmin (mmHg ms-1)
-1.0
-1.5
(m)
dP/dtmin rel. level
0.3
0.2
(n)
dP/dtmin delay (s)
0.085
0.080
(o)
dP/dtd delay (s)
0.156
0.152
(p)
dP/dtd - dP/dtmax (mmHg)
-10
-12
(q)
dP/dtd - dP/dtmin (mmHg)
-2
-4
(r)
93
96
99
Systolic blood pressure (mmHg)

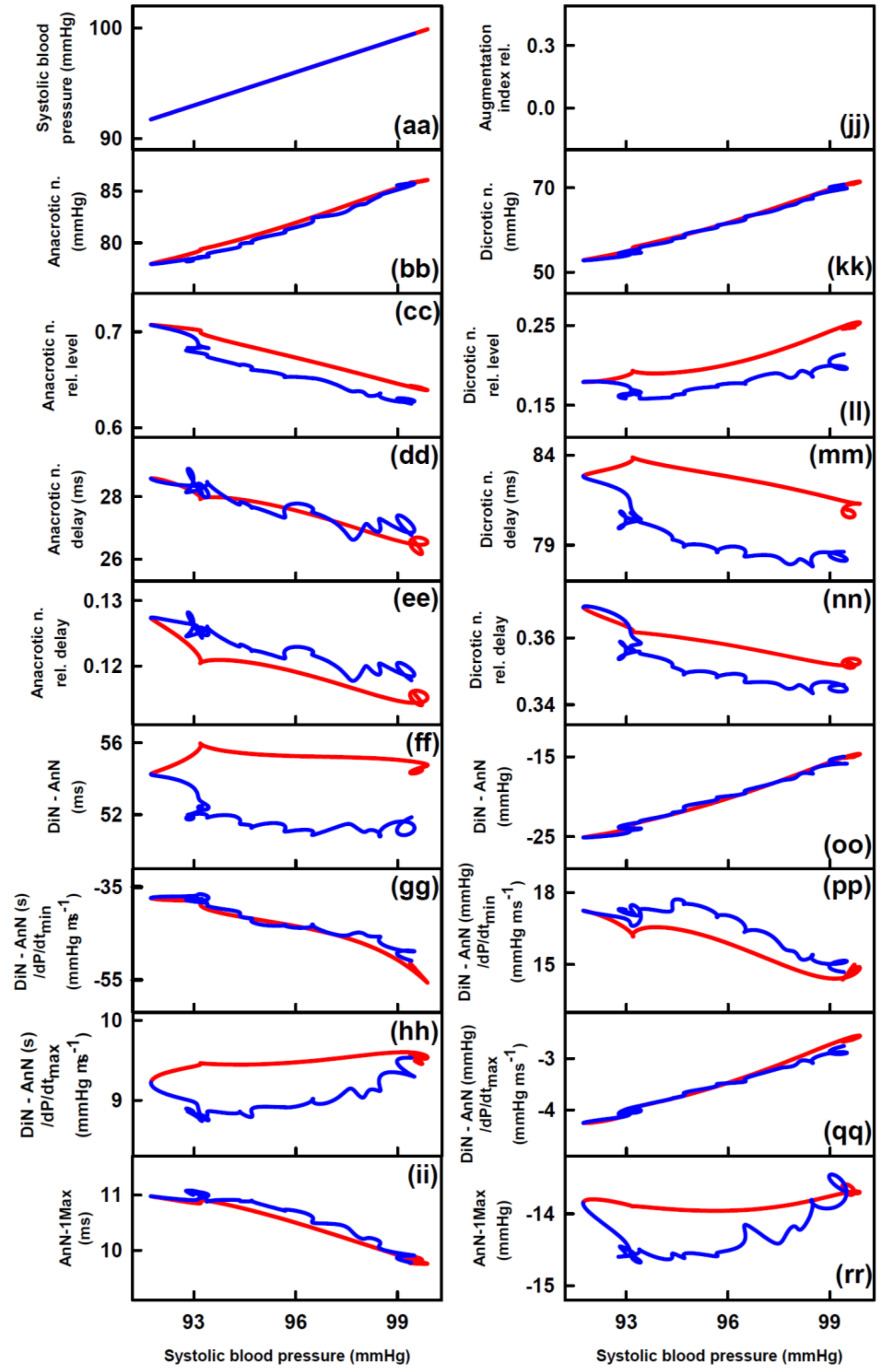

Figure S2C Relationships of HPs to systolic BP after administrations of 32 nmol kg^{-1} GSNO. The red line represents the decrease of the systolic BP from the control BP to the lowest BP and blue line represents the increase of the systolic BP from the lowest systolic BP to the control systolic BP (Figure 3A(a) or 3A(aa)).

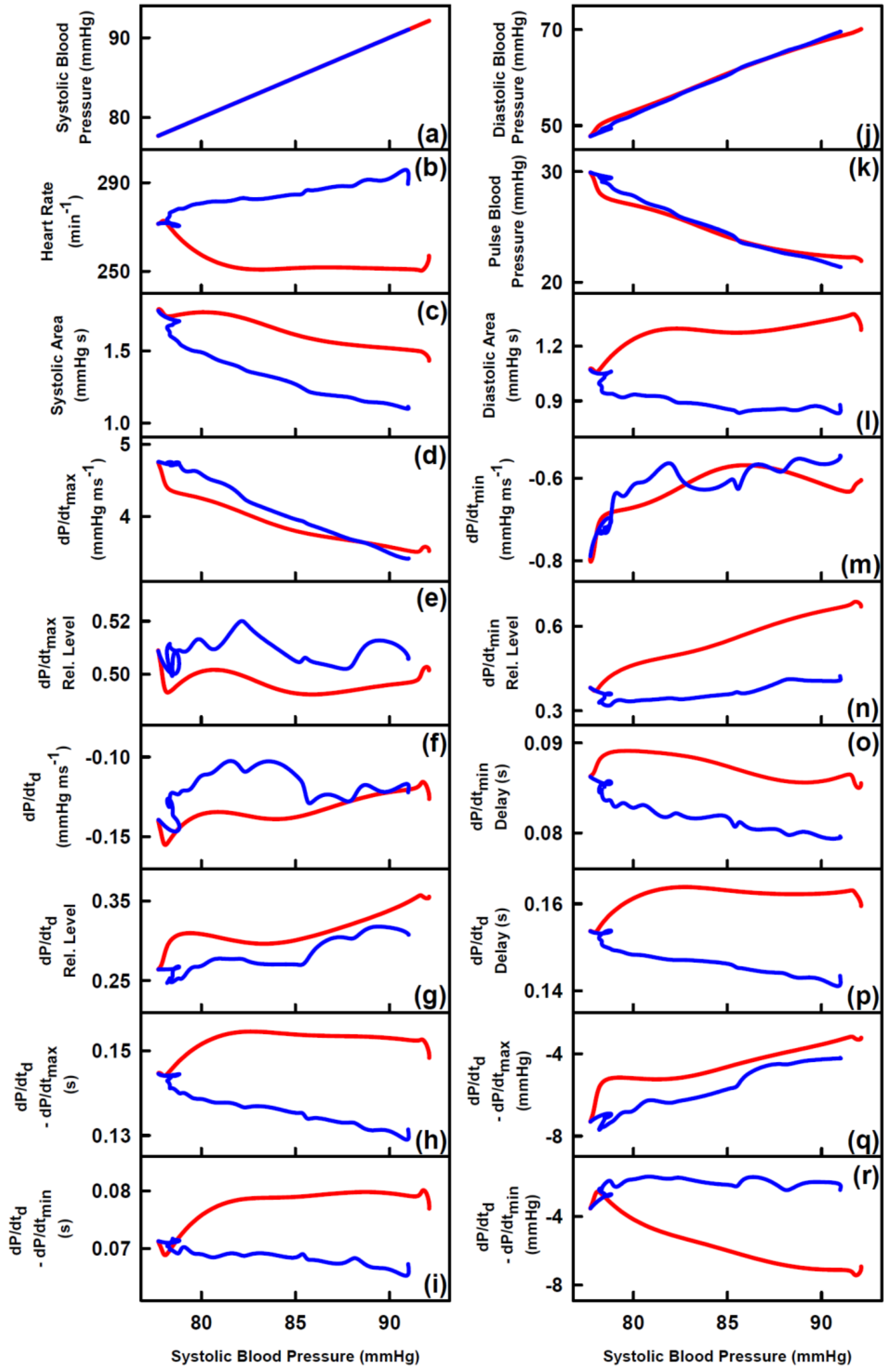
Systolic Blood Pressure (mmHg)
Heart Rate (min-1)
Systolic Area (mmHg s)
dP/dtmax (mmHg ms-1)
dP/dtmax Rel. Level
dP/dtd (mmHg ms-1)
dP/dtd Rel. Level
dP/dtd - dP/dtmax (s)
dP/dtd - dP/dtmin (s)
Diastolic Blood Pressure (mmHg)
Pulse Blood Pressure (mmHg)
Diastolic Area (mmHg s)
dP/dtmin (mmHg ms-1)
dP/dtmin Rel. Level
dP/dtmin Delay (s)
dP/dtd Delay (s)
dP/dtd - dP/dtmax (mmHg)
dP/dtd - dP/dtmin (mmHg)
(a) (b) (c) (d) (e) (f) (g) (h) (i) (j) (k) (l) (m) (n) (o) (p) (q) (r)
Systolic Blood Pressure (mmHg)
Systolic Blood Pressure (mmHg)

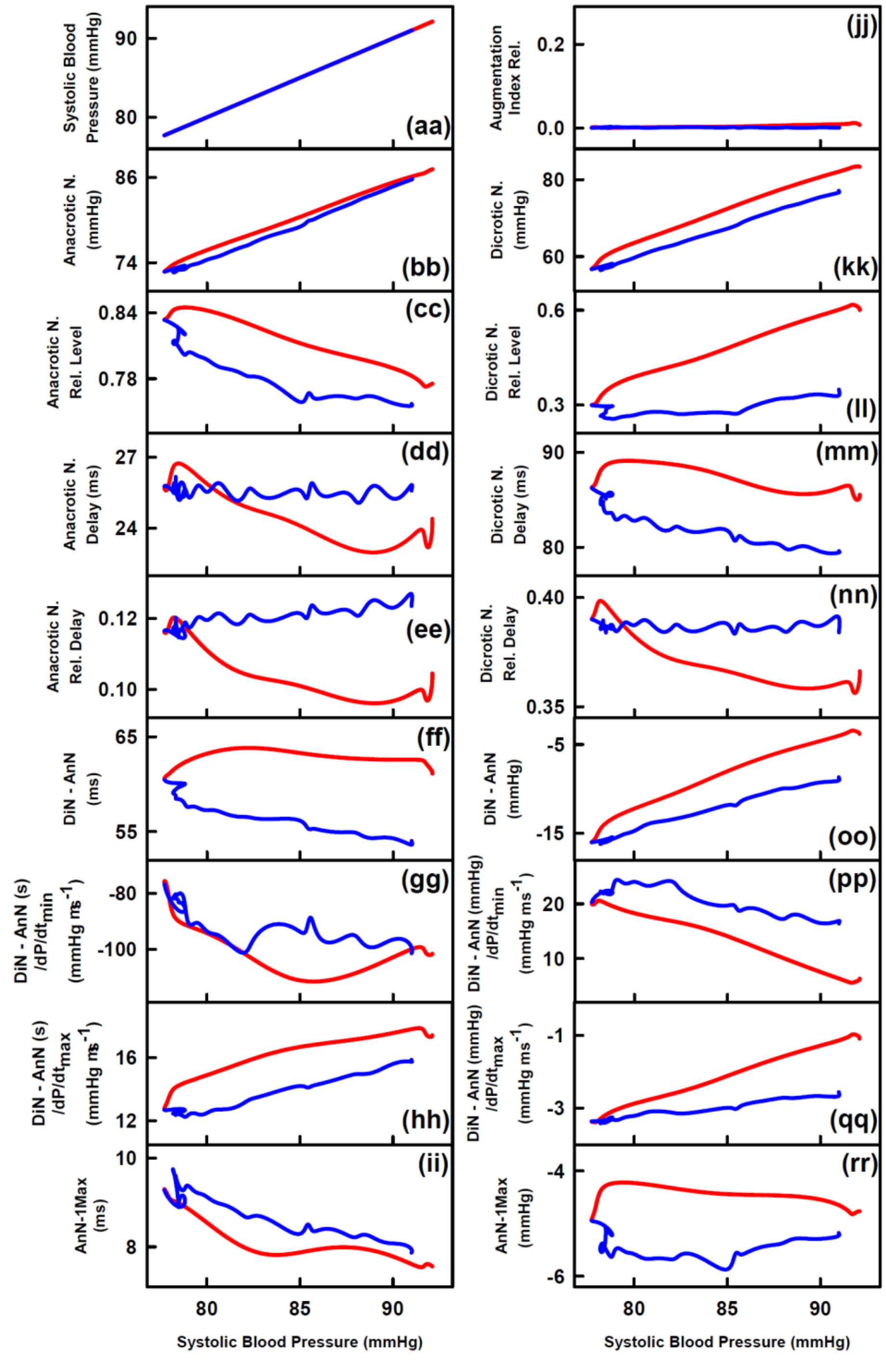

Figure S2D Relationships of HPs to systolic BP after administrations of 32 nmol kg^{-1} GSNO. The red line represents the decrease of the systolic BP from the control BP to the lowest BP and blue line represents the increase of the systolic BP from the lowest systolic BP to the control systolic BP (Figure 3A(a) or 3A(aa)).

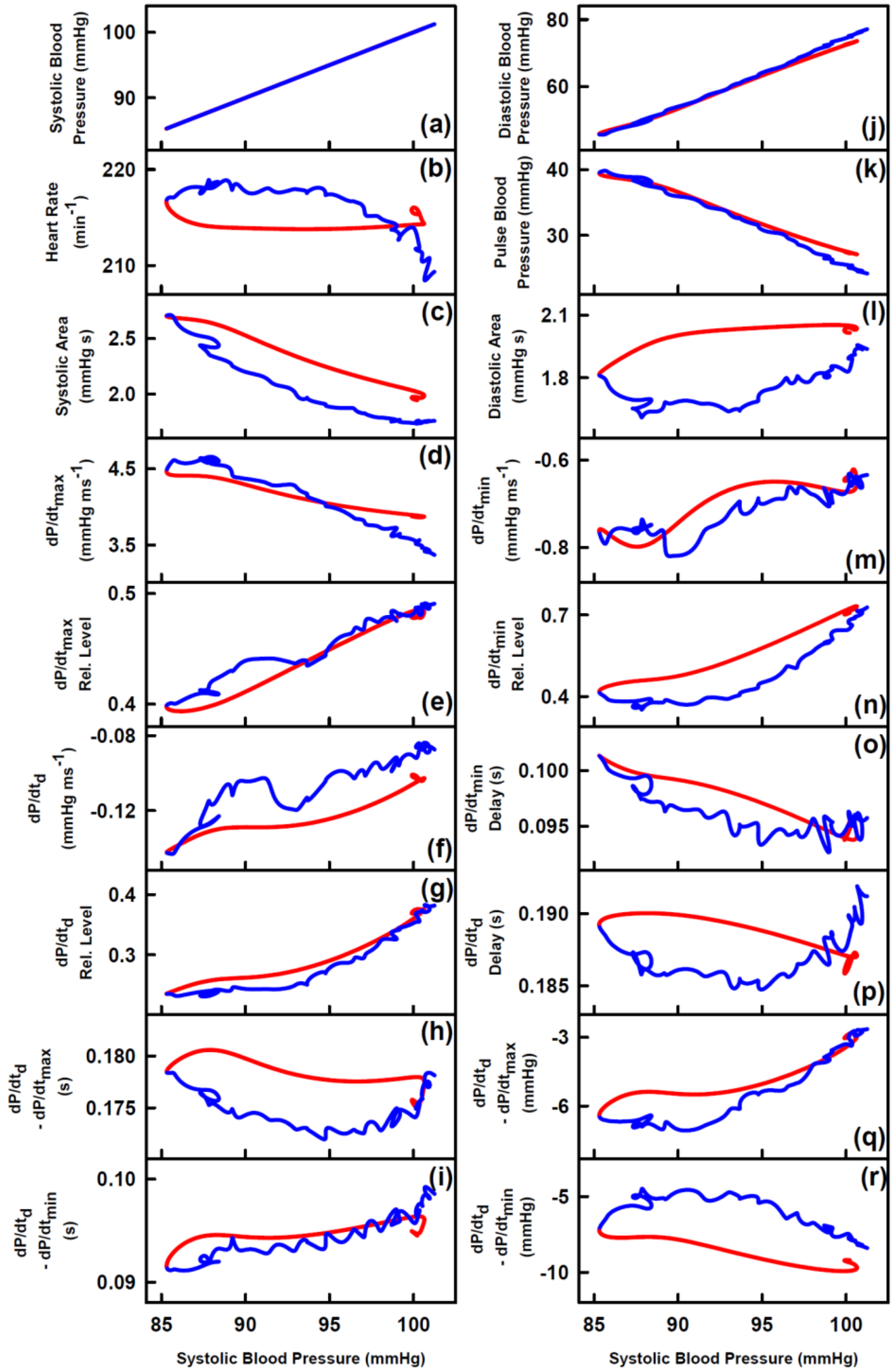

Systolic Blood Pressure (mmHg)
Heart Rate (min-1)
Systolic Area (mmHg s)
dP/dtmax (mmHg ms-1)
dP/dtmax Rel. Level
dP/dtd (mmHg ms-1)
dP/dtd Rel. Level
dP/dtd - dP/dtmax (s)
dP/dtd - dP/dtmin (s)
Diastolic Blood Pressure (mmHg)
Pulse Blood Pressure (mmHg)
Diastolic Area (mmHg s)
dP/dtmin (mmHg ms-1)
dP/dtmin Rel. Level
dP/dtmin Delay (s)
dP/dtd Delay (s)
dP/dtd - dP/dtmax (mmHg)
dP/dtd - dP/dtmin (mmHg)
(a)
(b)
(c)
(d)
(e)
(f)
(g)
(h)
(i)
(j)
(k)
(l)
(m)
(n)
(o)
(p)
(q)
(r)
85
90
95
100
Systolic Blood Pressure (mmHg)

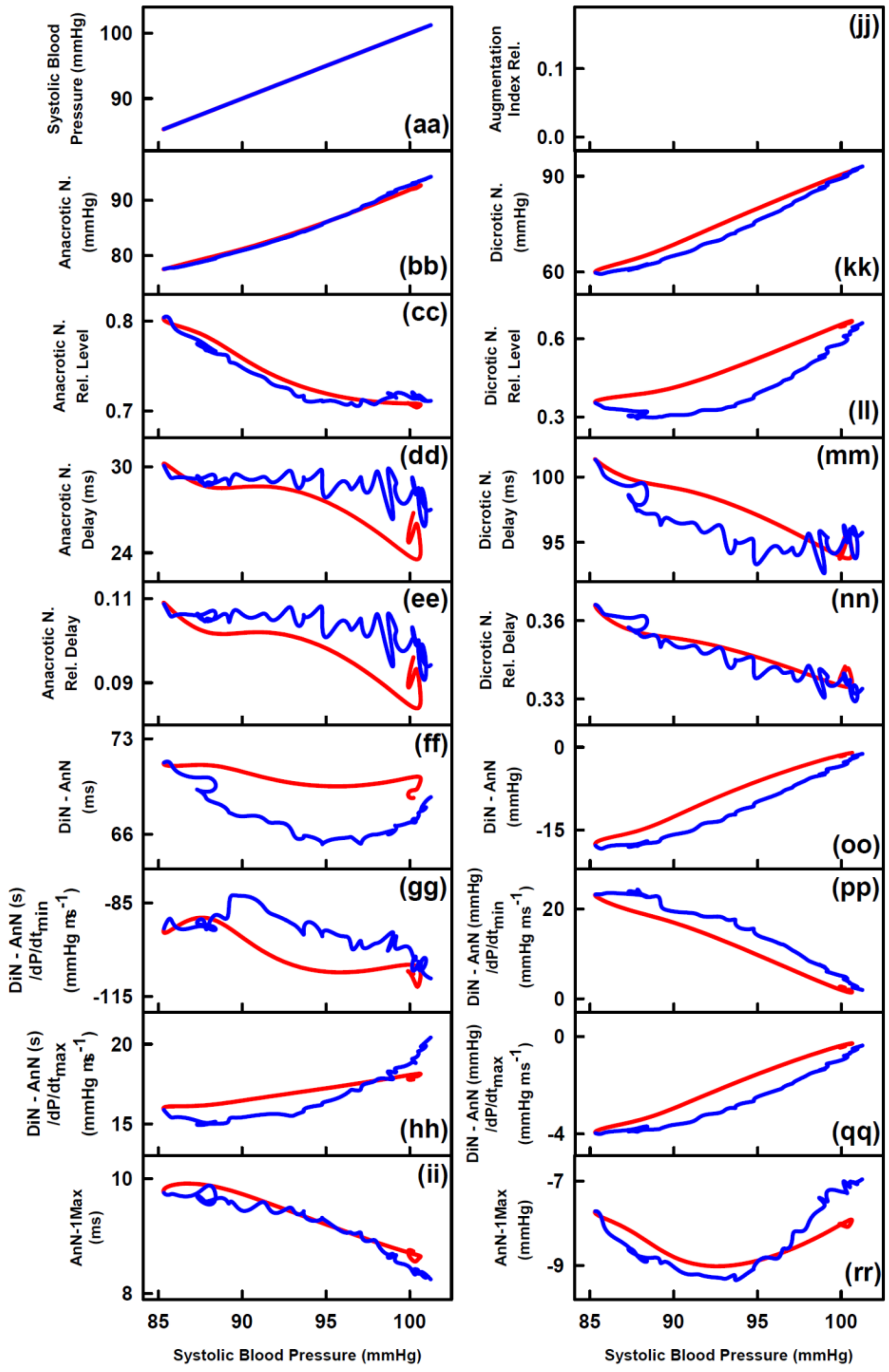

Figure S2E Relationships of HPs to systolic BP after administrations of 32 nmol kg^{-1} GSNO. The red line represents the decrease of the systolic BP from the control BP to the lowest BP and blue line represents the increase of the systolic BP from the lowest systolic BP to the control systolic BP (Figure 3A(a) or 3A(aa)).

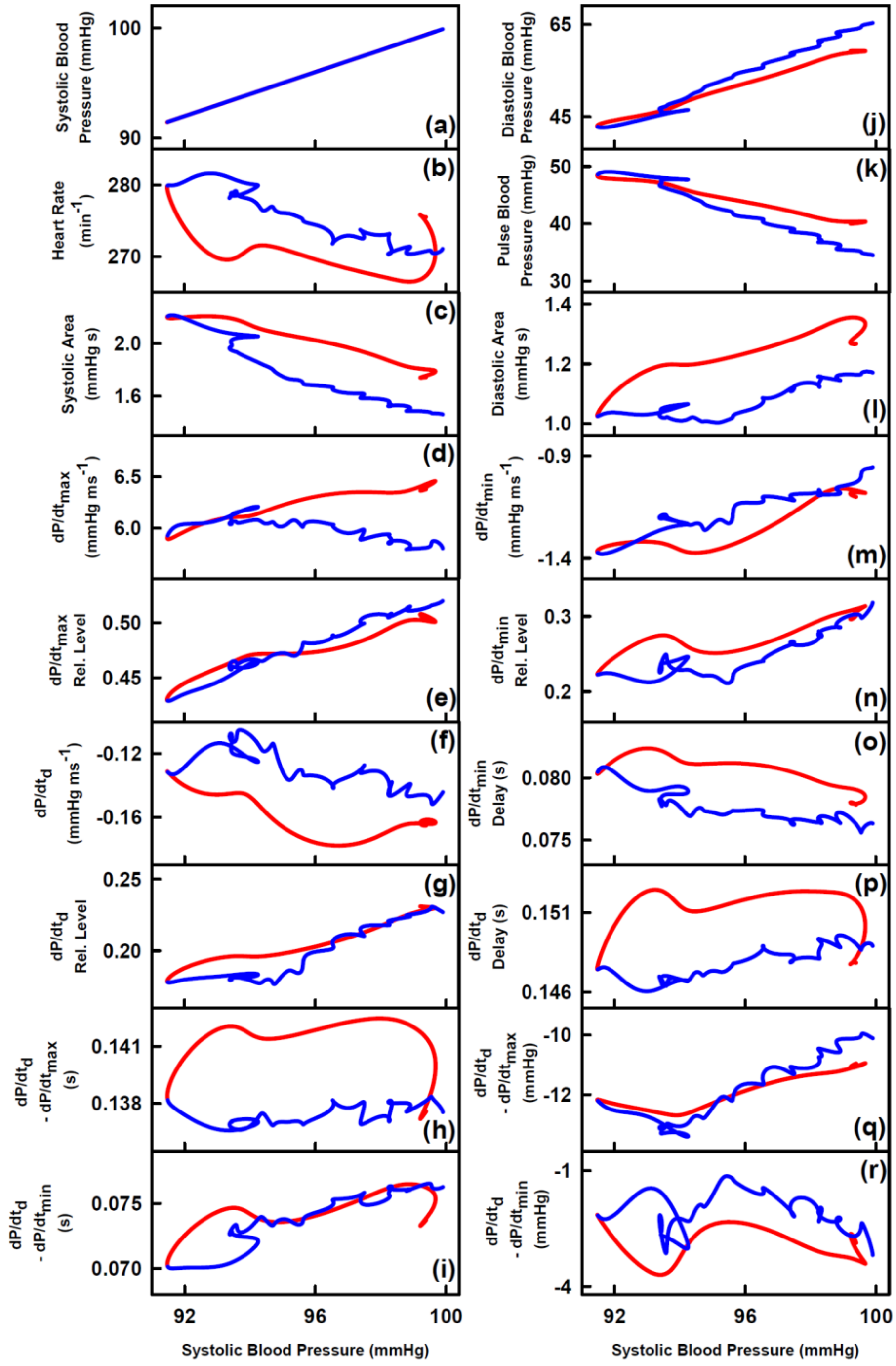

Systolic Blood Pressure (mmHg)
Heart Rate (min-1)
Systolic Area (mmHg s)
dP/dtmax (mmHg ms-1)
dP/dtmax Rel. Level
dP/dtd (mmHg ms-1)
dP/dtd Rel. Level
dP/dtd - dP/dtmax (s)
dP/dtd - dP/dtmin (s)
Diastolic Blood Pressure (mmHg)
Pulse Blood Pressure (mmHg)
Diastolic Area (mmHg s)
dP/dtmin (mmHg ms-1)
dP/dtmin Rel. Level
dP/dtmin Delay (s)
dP/dtd Delay (s)
dP/dtd - dP/dtmax (mmHg)
dP/dtd - dP/dtmin (mmHg)
(a) (b) (c) (d) (e) (f) (g) (h) (i)
(j) (k) (l) (m) (n) (o) (p) (q) (r)
Systolic Blood Pressure (mmHg)
Systolic Blood Pressure (mmHg)

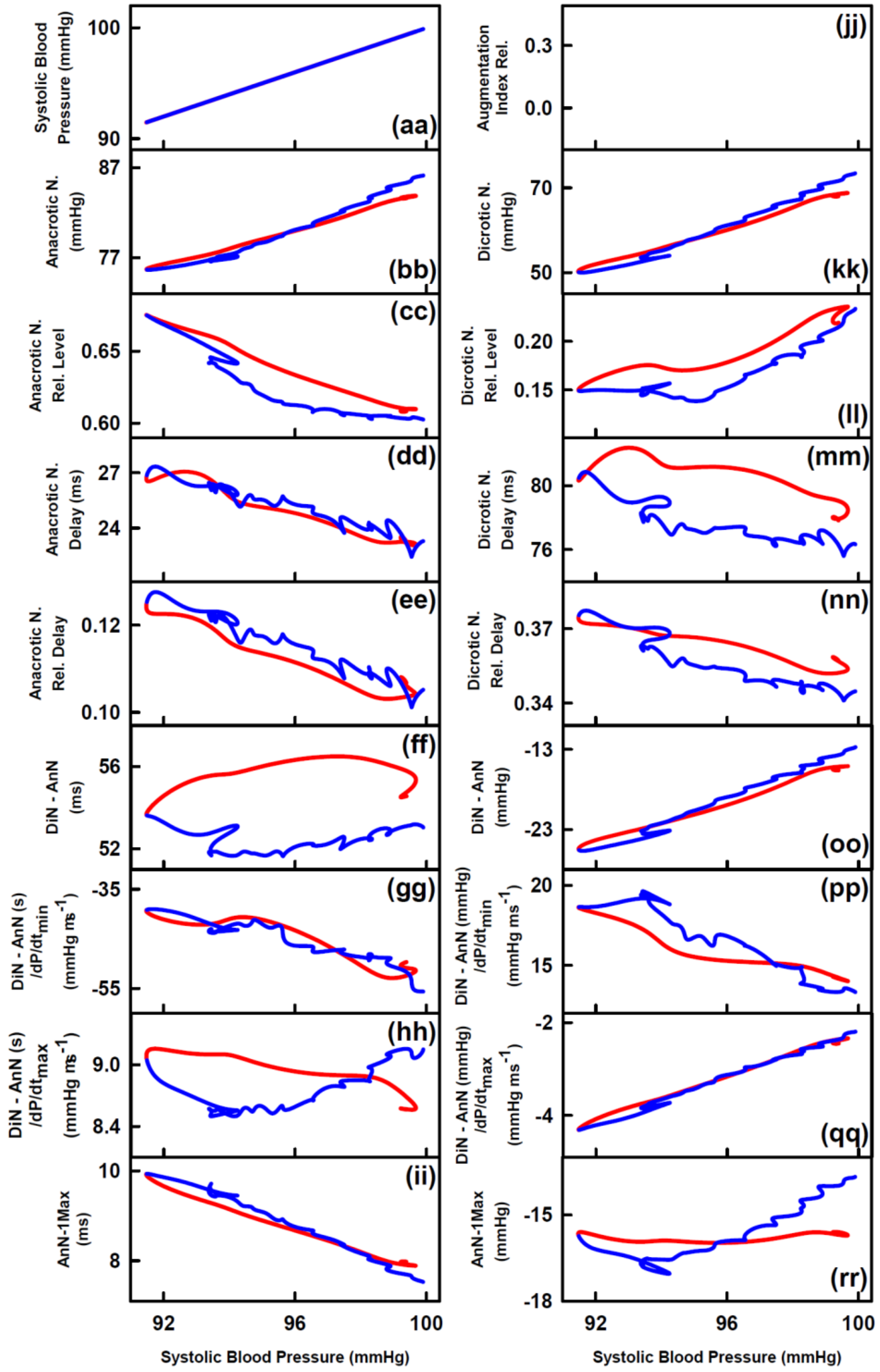

Figure S2F Relationships of HPs to systolic BP after administrations of 32 nmol kg^{-1} GSNO. The red line represents the decrease of the systolic BP from the control BP to the lowest BP and blue line represents the increase of the systolic BP from the lowest systolic BP to the control systolic BP (Figure 3A(a) or 3A(aa)).

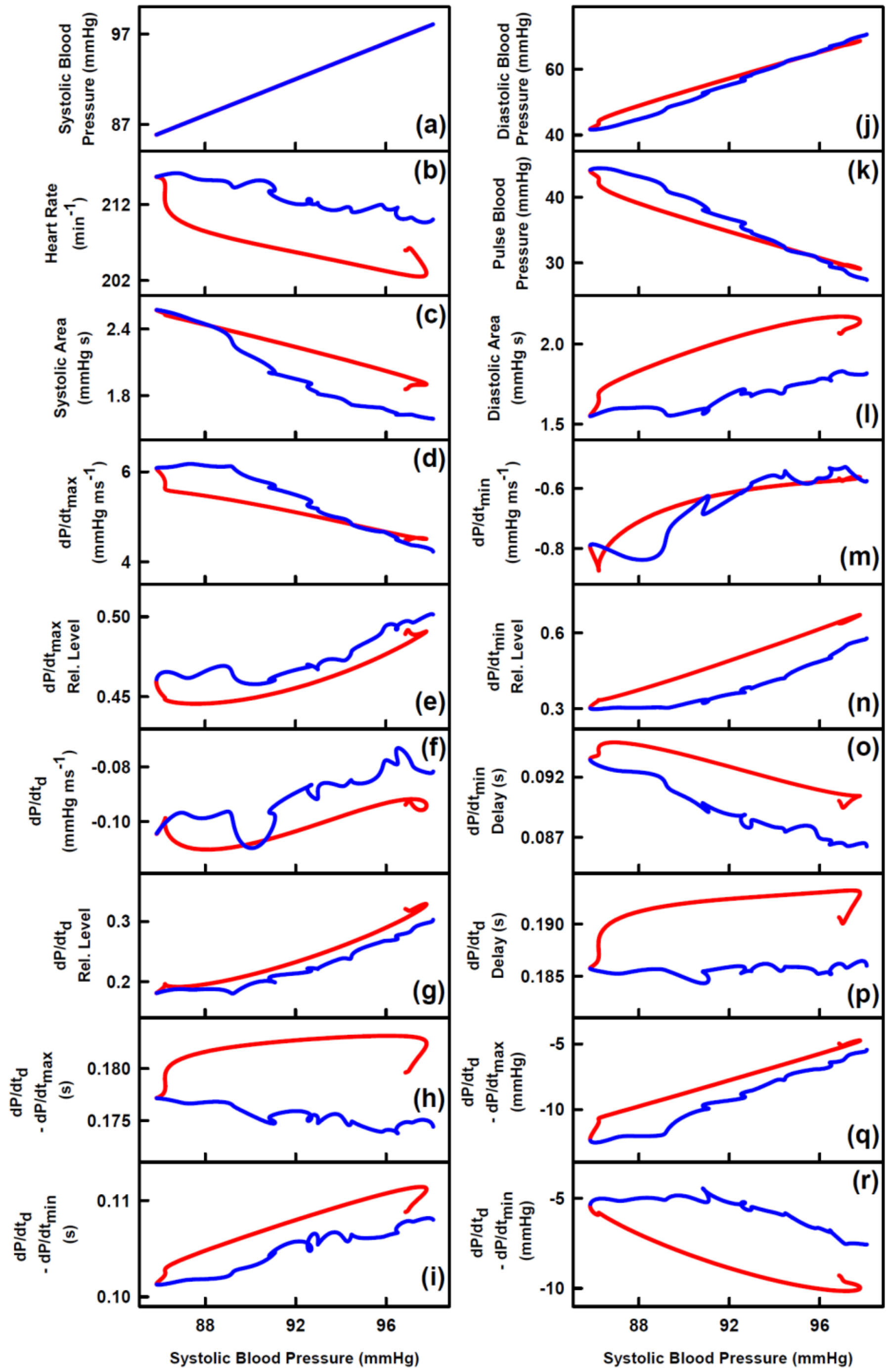

Systolic Blood Pressure (mmHg)
97
87
(a)
Heart Rate (min-1)
212
202
(b)
Systolic Area (mmHg s)
2.4
1.8
(c)
dP/dtmax (mmHg ms-1)
6
4
(d)
dP/dtmax Rel. Level
0.50
0.45
(e)
dP/dtd (mmHg ms-1)
-0.08
-0.10
(f)
dP/dtd Rel. Level
0.3
0.2
(g)
dP/dtd - dP/dtmax (s)
0.180
0.175
(h)
dP/dtd - dP/dtmin (s)
0.11
0.10
(i)
88
92
96
Systolic Blood Pressure (mmHg)
Diastolic Blood Pressure (mmHg)
60
40
(j)
Pulse Blood Pressure (mmHg)
40
30
(k)
Diastolic Area (mmHg s)
2.0
1.5
(l)
dP/dtmin (mmHg ms-1)
-0.6
-0.8
(m)
dP/dtmin Rel. Level
0.6
0.3
(n)
dP/dtmin Delay (s)
0.092
0.087
(o)
dP/dtd Delay (s)
0.190
0.185
(p)
dP/dtd - dP/dtmax (mmHg)
-5
-10
(q)
dP/dtd - dP/dtmin (mmHg)
-5
-10
(r)
88
92
96
Systolic Blood Pressure (mmHg)

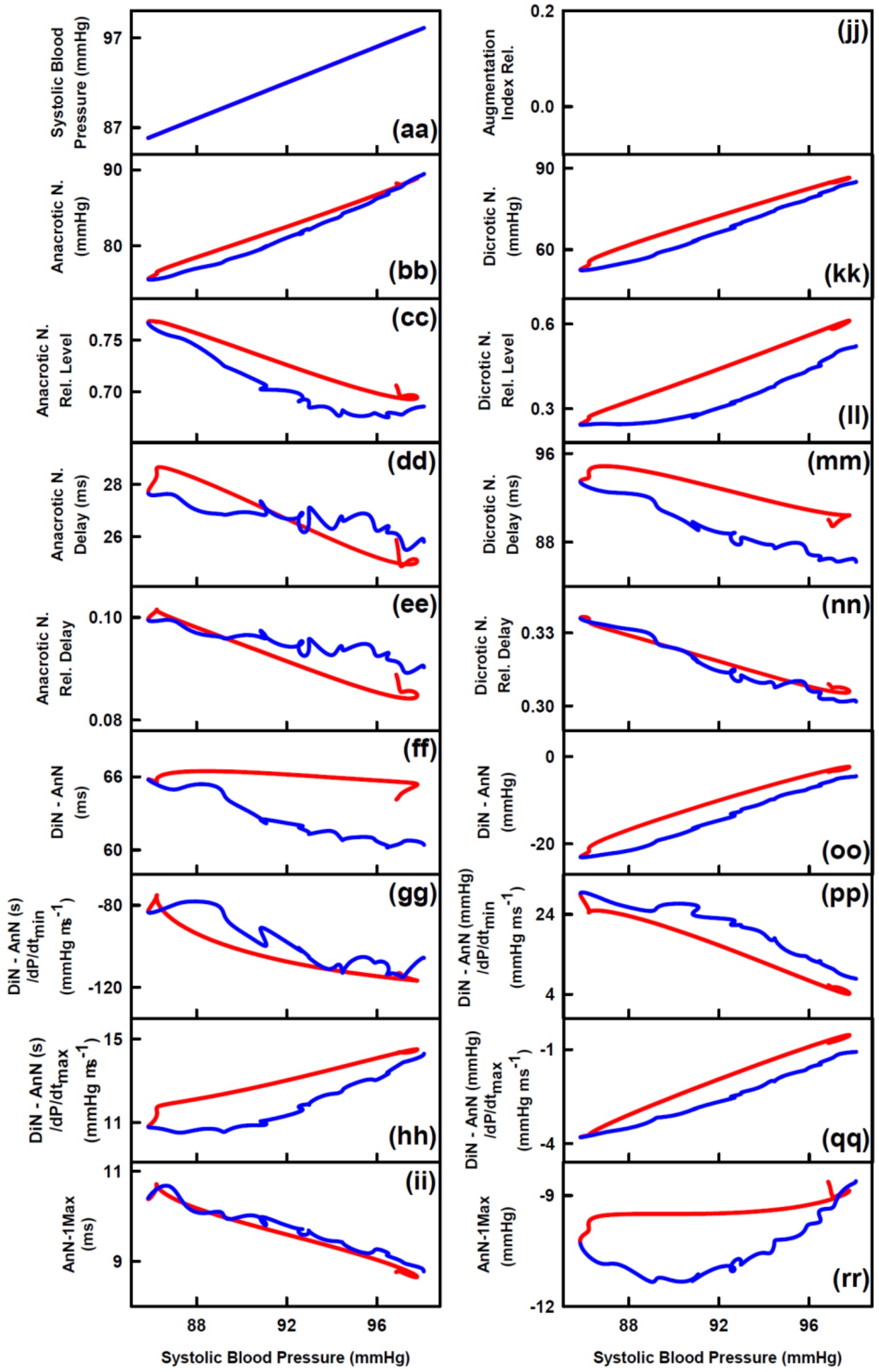

Figure S2G Relationships of HPs to systolic BP after administrations of 32 nmol kg^{-1} GSNO. The red line represents the decrease of the systolic BP from the control BP to the lowest BP and blue line represents the increase of the systolic BP from the lowest systolic BP to the control systolic BP (Figure 3A(a) or 3A(aa)).

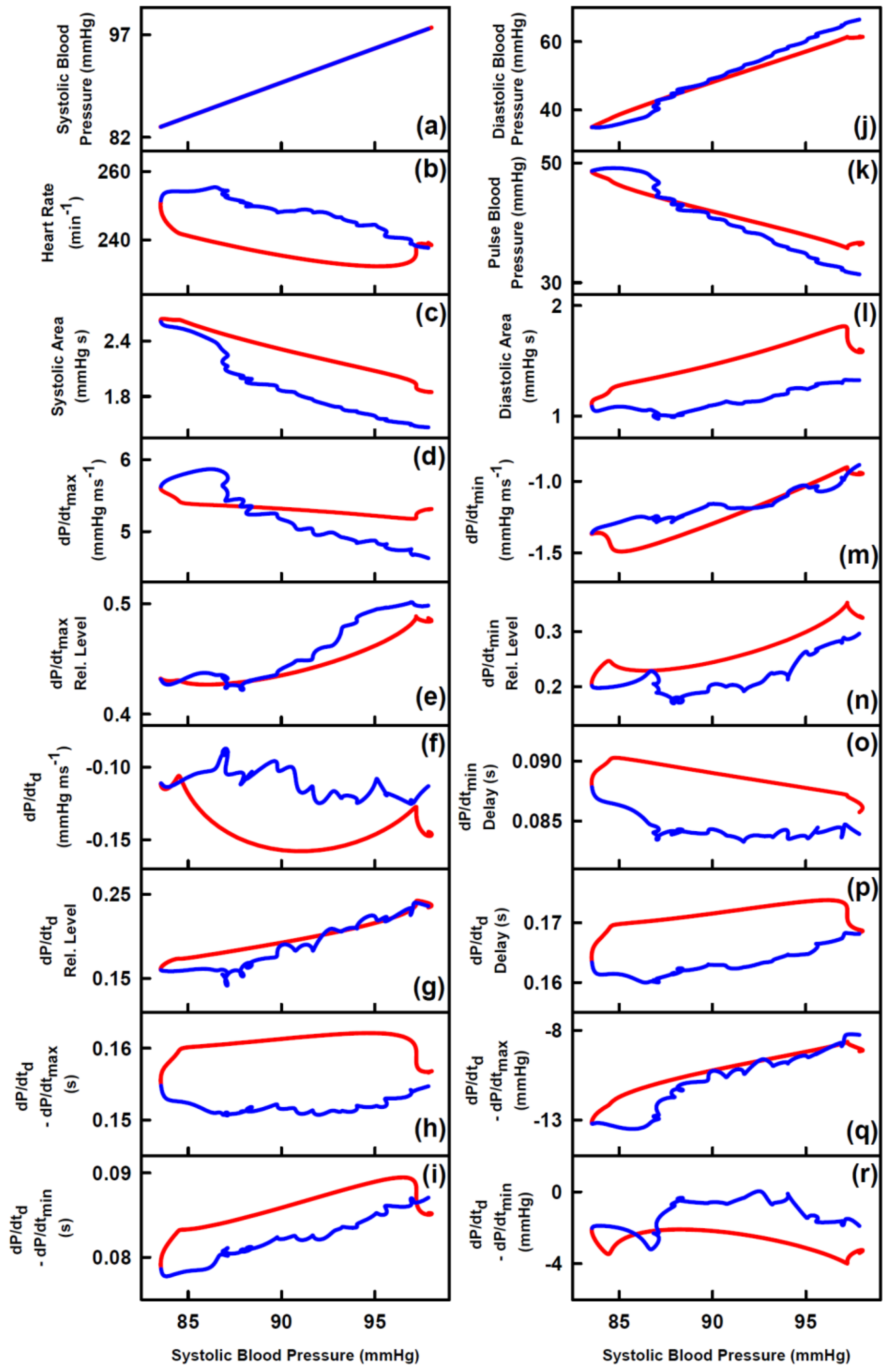
Systolic Blood Pressure (mmHg)
Heart Rate (min-1)
Systolic Area (mmHg s)
dP/dtmax (mmHg ms-1)
dP/dtmax Rel. Level
dP/dtd (mmHg ms-1)
dP/dtd Rel. Level
dP/dtd - dP/dtmax (s)
dP/dtd - dP/dtmin (s)
Diastolic Blood Pressure (mmHg)
Pulse Blood Pressure (mmHg)
Diastolic Area (mmHg s)
dP/dtmin (mmHg ms-1)
dP/dtmin Rel. Level
dP/dtmin Delay (s)
dP/dtd Delay (s)
dP/dtd - dP/dtmax (mmHg)
dP/dtd - dP/dtmin (mmHg)
(a)
(b)
(c)
(d)
(e)
(f)
(g)
(h)
(i)
(j)
(k)
(l)
(m)
(n)
(o)
(p)
(q)
(r)
Systolic Blood Pressure (mmHg)
Systolic Blood Pressure (mmHg)

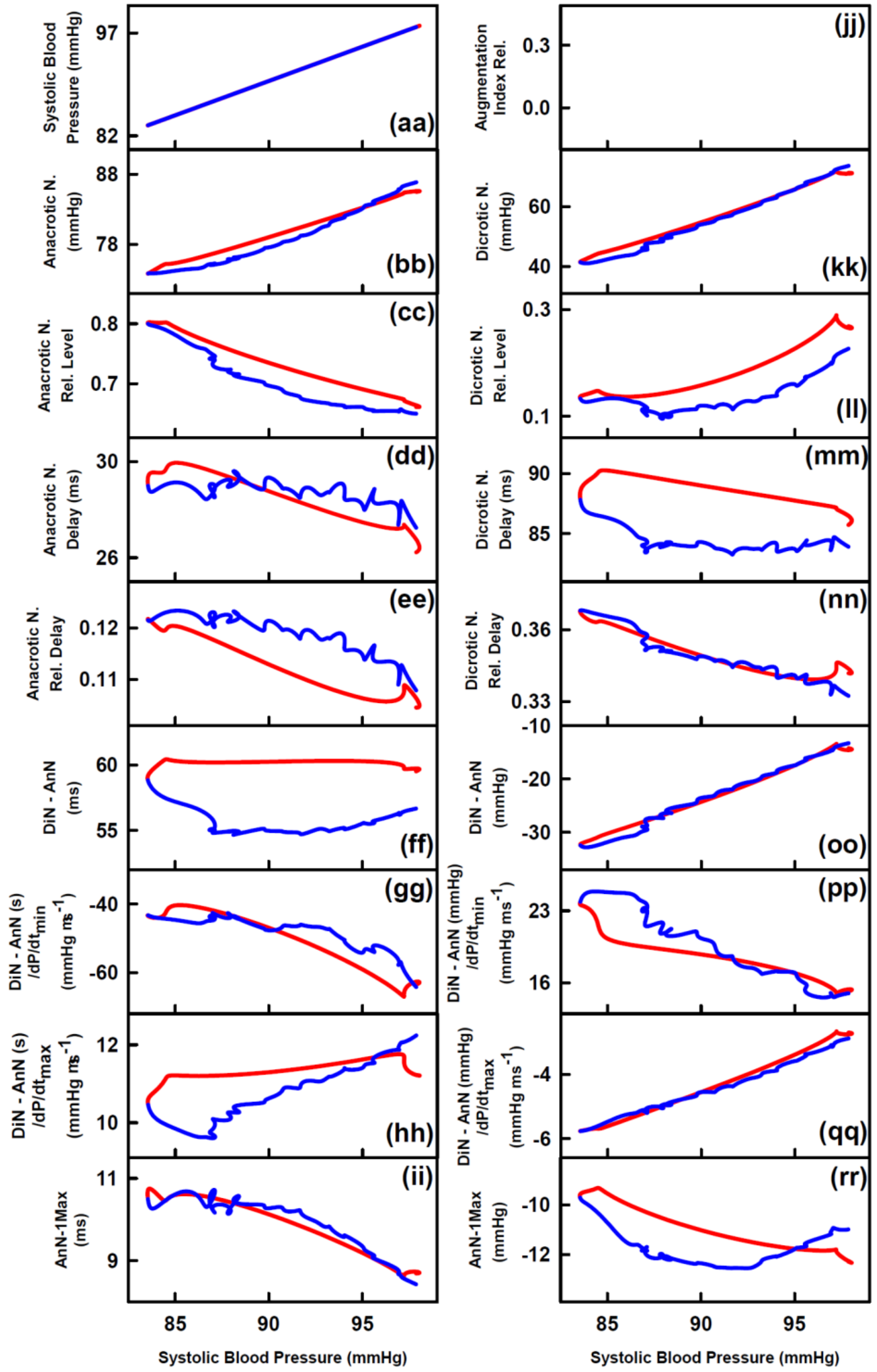

Figure S2H Relationships of HPs to systolic BP after administrations of 32 nmol kg^{-1} GSNO. The red line represents the decrease of the systolic BP from the control BP to the lowest BP and blue line represents the increase of the systolic BP from the lowest systolic BP to the control systolic BP (Figure 3A(a) or 3A(aa)).

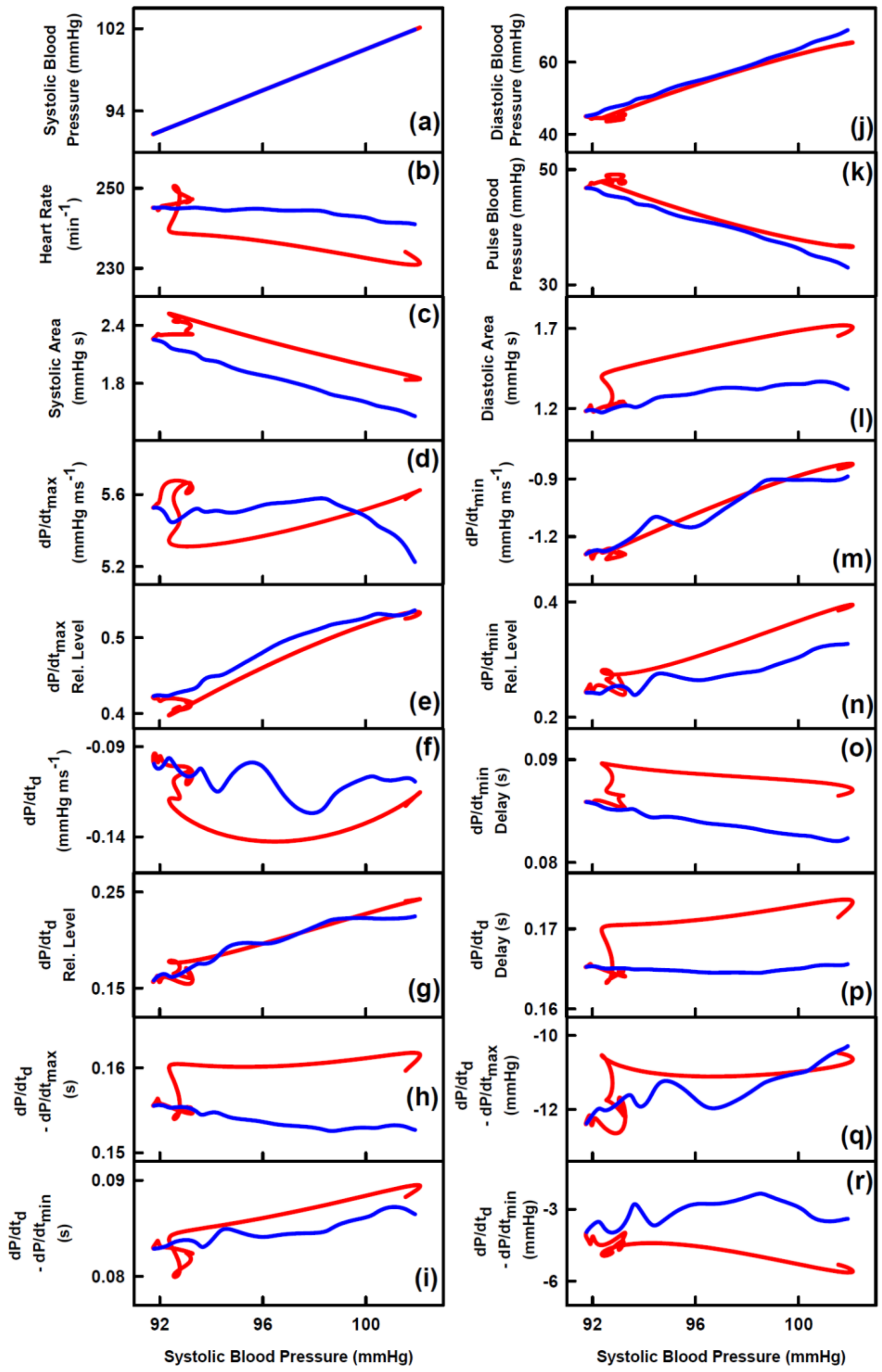

Systolic Blood Pressure (mmHg)
Heart Rate (min-1)
Systolic Area (mmHg s)
dP/dtmax (mmHg ms-1)
dP/dtmax Rel. Level
dP/dtd (mmHg ms-1)
dP/dtd Rel. Level
dP/dtd - dP/dtmax (s)
dP/dtd - dP/dtmin (s)
Diastolic Blood Pressure (mmHg)
Pulse Blood Pressure (mmHg)
Diastolic Area (mmHg s)
dP/dtmin (mmHg ms-1)
dP/dtmin Rel. Level
dP/dtmin Delay (s)
dP/dtd Delay (s)
dP/dtd - dP/dtmax (mmHg)
dP/dtd - dP/dtmin (mmHg)
(a)
(b)
(c)
(d)
(e)
(f)
(g)
(h)
(i)
(j)
(k)
(l)
(m)
(n)
(o)
(p)
(q)
(r)
Systolic Blood Pressure (mmHg)
Systolic Blood Pressure (mmHg)

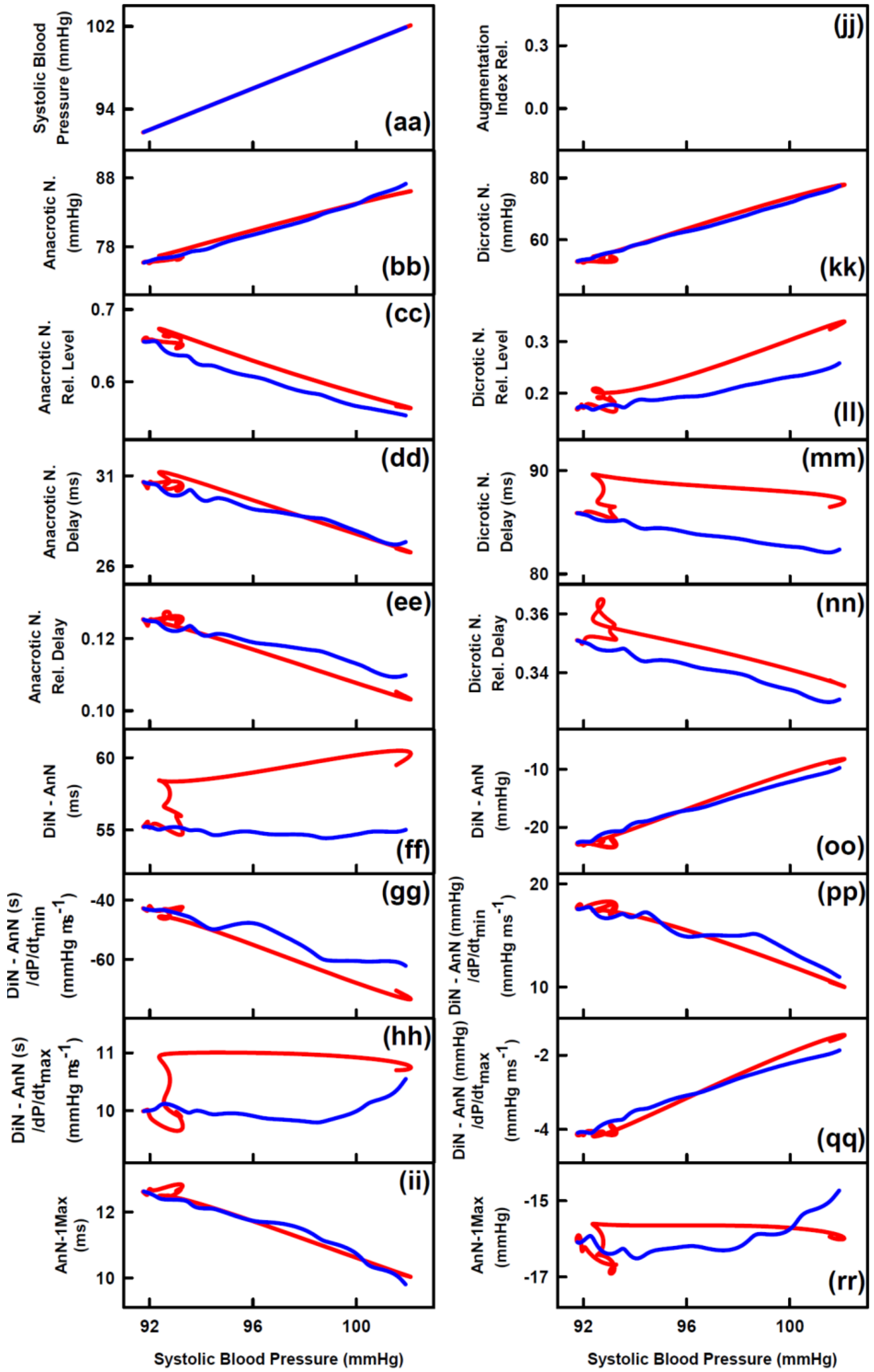

Figure S2I Relationships of HPs to systolic BP after administrations of 32 nmol kg^{-1} GSNO. The red line represents the decrease of the systolic BP from the control BP to the lowest BP and blue line represents the increase of the systolic BP from the lowest systolic BP to the control systolic BP (Figure 3A(a) or 3A(aa)).
